# A Stilbenoid Isorhapontigenin as a Potential Anti-Cancer Agent against Breast Cancer through Inhibiting Sphingosine Kinases/Tubulin Stabilization

**DOI:** 10.3390/cancers11121947

**Published:** 2019-12-05

**Authors:** Lalita Subedi, Mahesh Kumar Teli, Jae Hyuk Lee, Bhakta Prasad Gaire, Mi-hyun Kim, Sun Yeou Kim

**Affiliations:** College of Pharmacy, Gachon University, #191, Hambakmoero, Yeonsu-gu, Incheon 21936, Korea; subedilali@gmail.com (L.S.); maheshkumar.teli@gmail.com (M.K.T.); wogur6378@naver.com (J.H.L.); samarpanbp@gmail.com (B.P.G.); kmh0515@gachon.ac.kr (M.-h.K.)

**Keywords:** isorhapontigenin, breast cancer, SPHK1, SPHK2, tubulin destabilization, apoptosis, proliferation

## Abstract

Isorhapontigenin (ISO), a tetrahydroxylated stilbenoid, is an analog of resveratrol (Rsv). The various biological activities of Rsv and its derivatives have been previously reported in the context of both cancer and inflammation. However, the anti-cancer effect of ISO against breast cancer has not been well established, despite being an orally bioavailable dietary polyphenol. In this study, we determine the anti-cancer effects of ISO against breast cancer using MCF7, T47D, and MDA-MB-231 cell lines. We observed that ISO induces breast cancer cell death, cell cycle arrest, oxidative stress, and the inhibition of cell proliferation. Additionally, sphingosine kinase inhibition by ISO controlled tubulin polymerization and cancer cell growth by regulating MAPK/PI3K-mediated cell cycle arrest in MCF7 cells. Interestingly, SPHK1/2 gene silencing increased oxidative stress, cell death, and tubulin destabilization in MCF7 cells. This suggests that the anti-cancer effect of ISO can be regulated by SPHK/tubulin destabilization pathways. Overall, ISO successfully induced breast cancer cell death and cell growth arrest, suggesting this phytochemical is a better alternative for breast cancer treatment. Further studies in animal models could confirm the potency and usability of ISO over Rsv for targeting breast cancer, potentially posing an alternative candidate for improved therapy in the near future.

## 1. Introduction

Breast cancer is one of the most frequently diagnosed cancers among women worldwide [1]. Almost 15–20% of total breast cancers are covered by triple-negative breast cancer (TNBC), while the remaining are of non-TNBC [2]. Among breast cancer patients, non-white women are mostly affected by TNBC, whereas non-TNBC affects all women irrespective of race [3]. Drug resistance and cancer recurrence are the major unsolved problems concerning breast cancer therapy. The severe side effects of chemotherapy and delayed drug resistance further complicate the cancer treatment procedure [4]. Phytochemicals are the most desirable chemopreventive agents because of their greater safety profile. The dietary polyphenol resveratrol (trans -3,4′,5-trihydroxystilbene) (Rsv) was reported to have cancer-preventing effects, such as inhibiting breast cancer cell proliferation, metastasis, and growth, followed by inducing cancer cell death [5]. Rsv, alone or in combination with other phytochemicals such as melatonin and quercetin, showed strong anti-cancer effects against both in vitro and in vivo experimental models of breast cancer, indicating the plausible therapeutic potential of Rsv for breast cancer management [6].

Rsv-mediated sirtuin1 (Sirt1) activation, sphingosine kinase (SPHK) inhibition, and tubulin destabilization are the primary targets for treatment strategies against several cancer types [7,8,9]. A considerable body of literature has suggested the strong anti-cancer effect of Sirt1 activator Rsv [10,11], despite few reports on the cancer-promoting activity of Sirt1 activation. Rsv inhibited cell growth and proliferation of TNBC as well as non-TNBC cells [12]. Rsv also reduced HepG2 cancer cell migration and proliferation through Sirt1 activation [13]. YK-3-237 deacetylates mutant p53 through Sirt1 activation resulting in the inhibition of the TNBC cancer cell proliferation [14]. These aforementioned studies collectively indicate the strong anti-cancer effects of Sirt1 activation. SPHKs are the enzymes required for S1P formation [15]. SPHKs and S1P are not only involved in cancer metastasis and prognosis, but they are also associated with drug resistance [16,17,18,19,20]. S1P can also promote cancer cell proliferation, migration, survival, angiogenesis/lymphangiogenesis, and immune responses, indicating its role as a cancerous agent [21]. Since SPHK1 and SPHK2 have equipotent effects in cancer metastasis and resistance, inhibiting these enzymes could decrease cancer cell growth and prevent cancer cell resistance [22,23]. This notion was further supported by the strong anti-cancer effect of the SPHK inhibitor SKI-178 against myeloid leukemia through its ability to promote microtubule disruption [24], indicating a close link between SPHK and tubulin, though this relationship has not been well elucidated. Altering tubulin structure integrity is an important target for cancer therapy, and was transparently explored previously via studying microtubule stabilizers and destabilizers, such as paclitaxel and vinca, respectively. These microtubule stabilizers and destabilizers can affect microtubule dynamics and hence show anti-cancer potency [25]. More importantly, Rsv is not only an SPHK inhibitor, but it is also a known tubulin destabilizer [9,26]. Therefore, the anti-cancer effects of Rsv are mediated through Sirt1 activation, SPHK inhibition, and tubulin destabilization. 

Despite the promising effects of Rsv against breast cancer, its poor bioavailability by rapid degradation through fast metabolism limits its use and efficacy [27]. Therefore, the preparation of Rsv in a combination form or identifying another Rsv analog with improved pharmacokinetic (PK) profile could result in the development of novel therapeutic agents for breast cancer treatment [28,29]. Recently, more interest has been generated regarding Rsv derivatives that have greater bioavailability than Rsv, ideally activating the same or different targets [30]. Among Rsv derivatives, isorhapontigenin (ISO), one of the bioavailable dietary stilbene, could also have promising therapeutic potential for breast cancer treatment, as stilbenoids have been previously reported for their anti-cancer activity through altering DNA methylation in breast cancer cells [31]. ISO is present in diverse natural resources, including different types of berries (blueberries), grapes, rhubarb, and red wines [32]. ISO is reported to have an antioxidant effect higher than that of vitamin E [33]. ISO has anti-allergic, antiviral, and cardioprotective effects [34,35,36]. Importantly, previous research has indicated that ISO could have better bioactivity than that of Rsv. Yeo et al. revealed that ISO suppresses airways epithelial cell inflammation, and this effect is comparatively higher than that of Rsv alone [37]. This suggests the greater potency of ISO compared to Rsv. Considering the health-promoting activities of ISO, Dai et al. performed a pre-clinical pharmacokinetic, metabolic analysis and reported that ISO had a good PK profile than that of Rsv [38]. Being an Rsv analog with a higher PK profile could make ISO a better dietary alternative for treating cancer. In fact, Fang et al. isolated ISO from the Chinese herbal medicine *Gnetum cleistostachyum* and observed the anti-cancer effect of this compound against bladder cancer [39]. Previous reports also suggested the anti-cancer effects ISO in various cancers including lung cancer, pancreatic cancer, colon cancer, and gastric cancer [39]. In addition, the anti-cancer effect of ISO against invasive bladder cancer was reported through cyclin D1 inhibition [39]. Cyclin D1 is extensively increased in breast cancer cells [40], indicating the possible anti-cancer effects of ISO against breast cancer cell lines. In addition, a recent report suggested the anticancer effects of ISO in TNBC cells through Nrf2-mediated pathways [41]. In this study, we aim to determine the anti-cancer effects of ISO against breast cancer cell survival and proliferation, possibly through regulating SPHKs, tubulin destabilization and Sirt1 activation. 

## 2. Materials and Methods

### 2.1. Reagents

Fetal bovine serum (FBS), penicillin-streptomycin (PS), and Dulbecco’s modified Eagle’s medium (DMEM) were purchased from Invitrogen (Carlsbad, CA, USA). Trypsin EDTA was bought from Gibco (Waltham, MA, USA). Isorhapontigenin was purchased from Sigma Chemical (St. Louis, MO, USA). Enzyme-linked immune sorbent assay (ELISA) development kits, tumor necrosis factor alpha (TNF-α), interleukin-6 (IL-6), and interleukin (IL-1β) were acquired from R&D Systems (Minneapolis, MN, USA). The primary antibodies α-tubulin, β-tubulin, SPHK1, SPHK2, PARP, caspase-3, caspase-9, p38, pp38, JNK, pJNK, ERK, and pERK were purchased from Cell Signaling (Beverly, MA, USA). Secondary antibodies for Sirt1, Bax, Bcl2, cytochrome-C, and GAPDH were purchased from Santa Cruz Technology. MCF7, T47D, and MDA-MB-231 cells were purchased from the Korean Cell Line Bank. 3-[4,5-Dimethyl-2-thiazolyl]-2,5-diphenyl-2-tetrazolium bromide (MTT) powder, RNase-A, propidium iodide, and DCFDA were purchased from Sigma-Aldrich (St. Louis, MO, USA). The annexin V-FITC apoptosis detection kit and trypan blue were purchased from R and D Systems. 

### 2.2. Cell Culture 

In this study, MCF7 and T47D cells were used as a representative cell for non-TNBCs, while MDA-MB-231 cells were used as a representative cell TNBCs. MCF7 cells were maintained in DMEM while T47D and MDA-MB-231 cells were maintained in RPMI medium. DMEM and RPMI medium were supplemented with 10% heat-inactivated FBS and 1% PS. Cells were stored in an incubator at 37 °C and 5% CO_2_. Once the cell confluence was almost 80–90%, cells were subcultured and maintained. Cells were seeded in 96- or 24-well plates with the desired quantity of cells, as per the experimental protocol [42]. After 24 h, seeded cells were treated with the desired compounds and incubated for the indicated time points depending upon the different experiments. Each treatment was performed in triplicate, and untreated cells with the same volume of treatment medium were used as a control group.

### 2.3. Western Blot Analysis

For the determination of protein expression, Western blot analysis was performed. Cells were lysed with pro-prep lysis buffer and incubated in ice, with occasional vortexing to enhance cell lysis. Cell lysates were centrifuged at 12,000× *g* for 20 min at 4 °C. Protein estimation was performed using Bradford reagent (Bio-Rad, Hercules, CA, USA). Proteins (30 µg) were separated in different percentages of SDS polyacrylamide gel electrophoresis (SDS-PAGE) depending on the protein size. The separated proteins in the gel were transferred to nitrocellulose membranes (Amersham Pharmacia Biotech, Buckinghamshire, UK), and blocked with 5% nonfat milk in Tris-buffered saline containing 0.1% Tween-20 for 1 h. The membrane was then incubated with respective primary antibodies at 4 °C overnight. The membrane was then incubated with respective secondary antibodies (ratio) for 2 h at RT. Protein bands were visualized using ECL reagents (Fujifilm, LAS-4000, Tokyo, Japan), and band intensity was determined using ImageJ software.

### 2.4. BrdU Proliferation Staining Assay and Immunofluorescence (IF) Labeling

The role of ISO in inhibiting breast cancer cell proliferation was evaluated using BrdU staining via immunofluorescence. MCF7 and MDA-MB-231 cells were seeded in a 24-well plate at a density of 1 × 10^4^ cells/well with glass cover slides of appropriate sizes and incubated overnight. Seeded cells were treated with ISO for the desired period of time (48 h) at the same time as BrdU co-treatment was performed. BrdU-stained cells were washed and then stained with DAPI for nuclear staining. They were then mounted with VECTA SHIELD mounting medium. Images were taken using a fluorescence microscope, as previously described [43].

### 2.5. Receptor X-Ray Structure

For performing the molecular docking study, the 3D coordinates of the X-ray crystallographic structure of tubulin complexed with combretastatins (PDB: 5LYJ) were selected among others for receptor grid generation. The protein structure was corrected by the protein preparation wizard (Schrödinger LLC., Portland, OR, USA.), and all hetero groups (except inhibitor and metal ion) were removed from the protein files. All water atoms and repeating extra chains were removed from the complex, and the structure was minimized before using the OPLS-2005 force field. Further, the ionization and tautomeric states of amino acid residues were corrected by H atom addition, as described previously [44].

### 2.6. Ligand Preparation

ISO, combretastatin, and Rsv were downloaded from internet websites (https://pubchem.ncbi.nlm.nih.gov) and prepared for 3D structure retrieval. The 3D structures of molecules were cleaned by the LigPrep module with the following parameters: (1) the OPLS-2005 force field was used; (2) the probable ionization states at pH 7.0 ± 2 were identified with the ionizer; (3) the salt was removed; (4) tautomers were generated for all states; (5) the 3D structure was used to determine chirality; (6) and one low-energy ring conformation per ligand was generated as described previously [45,46]. ISO, combretastatin, and Rsv were processed in a similar manner.

### 2.7. Structure-Based Virtual Screening

The prepared protein was used to make a grid for the docking experiment. The 15 Å grid box was centered at the ligand for the active site to accommodate a maximum ligand length. Default values for input partial charges and van der Waals scaling were used. The ligands were docked flexibly with a flip of the 5- and 6-membered rings. During docking, one pose per ligand was written out via post-docking minimization of the ligands [44]. Combretastatin was retrieved as mentioned above and docked using the extra precision (XP) mode of Glide. To evaluate the performance of the docking program, the docking conformation was aligned with the crystallized inhibitor conformation to check the root mean square deviation (RMSD). To identify the binding modes of ISO and Rsv against the above-processed tubulin, the XP mode of Glide was used with receptor flexibility. The default settings with no similarity scoring constraints were applied. The XP was used to perform automated docking with partial protein flexibility in the protein active site [47].

### 2.8. SPHK Enzyme Activity Assay

SPHK enzyme activity inhibition assay was performed in a 384-well plate. A 5-fold concentration of the compound was prepared in distilled water (DW), using 5% DMSO as a control. Firstly, 2 µL 5× compound, 2 µL 5× reaction buffer, and 2 µL 5× sphingosine kinase solution were mixed in each well and pre-incubated for 30 min. 2X ATP/ADP was used as a standard. Furthermore, 2 µL 5× sphingosine solution and 2 µL 5× ATP solution were added in each well except for the standard well, and the reaction was performed 2 h. ADP2 FI assay detection reagents were prepared, and 10 µL was added to every well. This was then incubated at RT for 1 h. The fluorescence intensity was measured at an excitation of 590 nm and an emission of 620 nm.

### 2.9. ROS Production Assay

To determine the effect of ISO on ROS production, cells were seeded in a 6-well plate at a density of 4 × 10^5^ cells/well and incubated overnight. Seeded cells were treated with ISO and Rsv for 1 h and stained with 30 μM 2′,7′-dichlorofluorescein diacetate (DCFH-DA) for 30 min at 37 °C to evaluate the intracellular level of ROS. Cells were washed with PBS and analyzed using the JULI imaging system and flow cytometry (FACSAriaIII; BD Biosciences, Franklin Lakes, NJ, USA), as previously described [48].

### 2.10. Measurement of Cell Viability (MTT Assay)

MCF7/T46D and MDA-MB-231 cells were seeded at a density of 5000 cells/well in a 96-well plate and incubated overnight. Seeded cells were treated with ISO and Rsv for 48 h. Most of the previous studies reported the highest anti-cancer efficacy of Rsv following 48 h of treatment [49,50,51]. Therefore, we chose this time point to determine the anti-cancer effects of ISO. Conditioned medium from the treated cells was removed, and the cells were incubated with 100 µL of MTT (0.5 mg/mL) solution for 1 h. Live cells were stained blue during this period. Once the cells were completely stained, the MTT solution was removed, and 200 µL of DMSO was added in each well. Blue-stained cells changed to a purple formazan, showing the number of viable cells. This was quantified calorimetrically by measuring the absorbance of the formazan solution at 570 nm in a microplate reader. Cell viability was calculated as a percentage of normal control (100%).

### 2.11. BrdU and Sulforhodamine B (SRB) Proliferation Assay

To determine the extent of cancer cell proliferation, BrdU assay was performed. MCF7/T46D and MDA-MB-231 cells were seeded in a 24-well plate at a density of 3 × 10^4^ cells/well and incubated overnight. Seeded cells were treated with ISO and Rsv together with BrdU (5 μM) solution and incubated for an additional 48 h. Treated cells were exposed to BrdU labeling solution, permeabilization solution, and DNase I. The extent of cell proliferation was analyzed using BrdU colorimetric assay kit (BD Bioscience, 559619) and quantified via colorimetric analysis by measuring the OD at 450 nm using a microplate reader.

We also used a sulforhodamine B (SRB) assay to determine cell proliferation as previously described [52]. For this, cells were seeded in a 96-well plate, treated with the appropriate compound, and incubated for 48 h. Adherent cells were fixed with 10% (*w*/*v*) trichloroacetic acid for 10 min and washed with running water. Cells were dried at RT, and stained with SRB for 30 min. The SRB solution was suctioned out, and excess SRB dye was removed by washing with 1% acetic acid. The protein-bound dye was dissolved by adding 10 mM Tris base solution, and the pink color formation was quantified by measuring absorbance (OD) at 510 nm using a microplate reader. SRB was calculated as a percentage of the untreated control.

### 2.12. LDH Assay for Cytotoxicity

To determine the cytotoxicity of tested compounds, a lactate dehydrogenase (LDH) LDH assay kit (Jiancheng Bio-Engineering, Nanjing, China) was used. The experiment was performed according to the manufacturer’s protocol with slight modifications. Briefly, MCF7/T46D and MDA-MB-231 cells were seeded in a 24-well plate at a density of 3 × 10^4^ cells/well and incubated overnight. Seeded cells were treated with ISO and Rsv for 48 h. Conditioned medium (50 µL) from the treated cells were mixed with equol volume (50 µL) of LDH mixture (containing LDH substrate), which catalyze the conversion of lactate to pyruvate, resulting in a brown-red color in basic solution. This color change was quantified colorimetrically by measuring the absorbance at a wavelength of 450 nm. 

### 2.13. Annexin V/PI Staining

An annexin V/PI apoptosis kit was used in accordance with the manufacturer’s instructions to quantify the percentage of cells undergoing apoptosis [53]. First, MCF7 cells were treated with the compounds and incubated for 48 h. Cells were collected, washed twice with cold PBS, and re-suspended in binding buffer at a concentration of 1 × 10^6^ cells/µL. Annexin V-FITC (5 µL) and PI (10 µL) were added, and the cells were incubated for 15 min at RT in the dark. Following incubation, 200 µL of binding buffer was added, and the cells were immediately analyzed by flow cytometry (FACSAriaIII; BD Biosciences, Franklin Lakes, NJ, USA). Flow cytometric analysis was performed using Cell Quest software (BD Biosciences). Annexin V+/PI− and annexin V−/PI+ cells were used to identify apoptotic and necrotic cells, respectively. The procedure was performed in triplicate for each sample.

### 2.14. Transfection with SPHK1 & SPHK2 siRNA 

MCF7 cells were seeded in a 6-well plate at a density of 3 × 10^5^ cells/well for 24 h followed by serum deprivation for 6h. SPHK1 and SPHK2 siRNA were prepared by mixing with lipofectamine RNA imax reagent at 1:1 ratio and transfected to the cells (200 µL; 50 nM final concentration) in serum and antibiotic-free medium. After 6 h of incubation, serum free medium was replaced with fresh medium containing serum, and cells were allowed to grow for additional 24 h. Cells were then treated with ISO for 48 h, and cell viability, ROS production, and protein expression were measured as previously described [54].

### 2.15. Measurement of TNF-α, IL-6, and IL-1β Production

MCF7/MDA-MB-231 cells were seeded in a 6-well plate at a density of 5 × 10^5^ cells/well and incubated overnight. Cells were treated with ISO and Rsv at different concentrations for 48 h. Conditioned medium from treated cells was collected to measure the level of secreted pro-inflammatory cytokines. The secreted levels of TNF-α, IL-6, and IL-1β were analyzed from conditioned medium using commercially available ELISA kits (human total TNF-α, IL-6, and IL-1β kit; R&D Systems) in accordance with the manufacturer’s instructions. 

### 2.16. Cell Cycle Analysis

MCF7 cells were seeded in a 6-well plate at a density of 5 × 10^5^ cells/well and treated with ISO and Rsv for 48 h. After completing the treatment, attached cells were washed with PBS, fixed with 70% ethanol, and kept at 4 °C until use for analysis. Cells were centrifuged at 300× *g*. The ethanol was removed and suspended cells were washed with PBS, followed by centrifugation. Cells were washed with 1 mL PBS, treated with 50 µL ribonuclease inhibitor, and incubated for 30 min at room temperature (RT). Propidium iodide (PI, 50 µL) was added to the cell mixture and incubated for 30 min at RT. Cell cycle analysis was quantified using (FACSAriaIII; BD Biosciences, Franklin Lakes, NJ, USA).

### 2.17. Cell Migration Assay

Breast cancer cells (MDA-MB-231) were seeded in 96-well plates (Essen Image Lock, Essen Bioscience, Ann Arbor, MI, USA) at 5.0 × 10^4^ cells/well, respectively. Scratch wound were prepared in the seeded cells by using a Wound Maker tool (Essen Bioscience, Ann Arbor, MI, USA). Cells were than washed with PBS, and co-incubated with different concentrations (5, 10, 20, and 40 μM) of ISO and Rsv (40 µM), respectively, in serum-free medium. Wound factors (relative wound density, wound confluence) were observed and photographed every 2 h for 36 h using IncuCyte ZOOM (Essen Bioscience).

### 2.18. Statistical Analysis

The results were evaluated using the Statistical Analysis System (GraphPad Prism 5, La Jolla, CA, USA). The results are presented as mean ± standard error of the mean (SEM), and all results are the mean of at least three independent experiments. A statistical comparison of different treatment groups was determined by one-way analysis of variance (ANOVA) followed by Tukey’s post-test. A value of *p* < 0.05 was considered statistically significant.

## 3. Results 

### 3.1. ISO Induces Tubulin Destabilization

**Figure 1:**
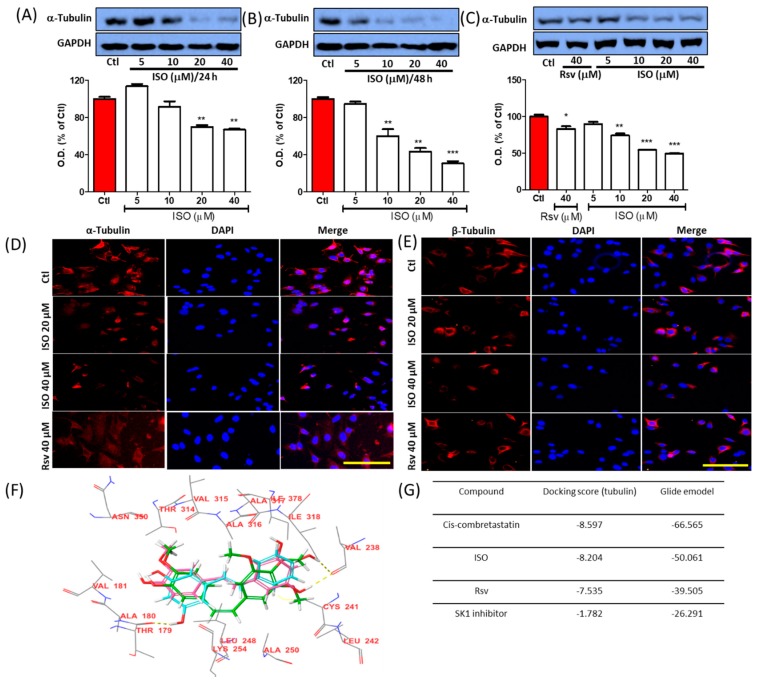
ISO treatment induced tubulin destabilization in MCF7 cells. MCF7 cells were treated with ISO and Rsv for the indicated time points and their roles in tubulin expression was determined using Western blot analysis. (**A**–**C**) Protein expression of tubulin and its densitometric analysis. GAPDH was used as a loading control. * *p* < 0.05, ** *p* < 0.01, and *** *p* < 0.001 indicate significant differences compared with untreated control group. (**D**,**E**) IF labeling of α and β-tubulin, Scale bars: 50 µm. (**F**) Tubulin interaction with ISO (pink) and resveratrol (blue). (**G**) Tubulin docking score.

Polymerized tubulin protein expression was determined through Western blot analysis as described previously [55]. ISO treatment showed clear inhibition of tubulin expression after both 24 and 48 h of treatment. Tubulin inhibition by ISO was compared with Rsv treatment. Though Rsv also slightly inhibited tubulin expression, ISO-mediated inhibition appeared much greater than Rsv at the same concentration (40 µM) (Figure 1A–C). Inhibition of such expression suggested that ISO can inhibit tubulin polymerization, and that this is the reason for inhibited tubulin expression. To confirm this, we performed IF staining for α- and β-tubulin (Figure 1D,E). Polymerization of α- and β-tubulins is an important step for microtubule formation. Imbalance between α- and β-tubulins levels disturb the tubulin polymerization, microtubule formation, and cellular homeostasis [56]. ISO showed significant potency for lowering the protein expression of polymerized α-tubulin at 24 and 48 h after treatment. This inhibitory effect of ISO was significantly higher than that of Rsv. In addition, intracellular expression of α- and β-tubulins were analyzed by IF assay and we observed the similar results. To further reaffirm this result, we performed the molecular docking study (Figure 1F,G). For this, we used combretastatin A-4 as a positive control because it is a potent anti-cancer agents having potential to induce cytotoxicity and inhibit tubulin binding. Rsv and ISO have structural similarity with cis-combretastatin, and demonstrate a similar binding pose in the colchicine-binding site of tubulin, indicating their roles in inhibiting tubulin polymerization. Previous reports have suggested that Rsv/its derivatives decreases tubulin polymerization [26,57], but in this study, we demonstrated the higher potency of ISO for lowering tubulin polymerization (Figure 1F,G). Therefore, tubulin destabilization could be a major mechanistic target of ISO for its anti-cancer effects in breast cancer. 

### 3.2. ISO Alters the Activity and Expression of Sphingosine Kinases (SPHK) in MCF7 Cells

**Figure 2:**
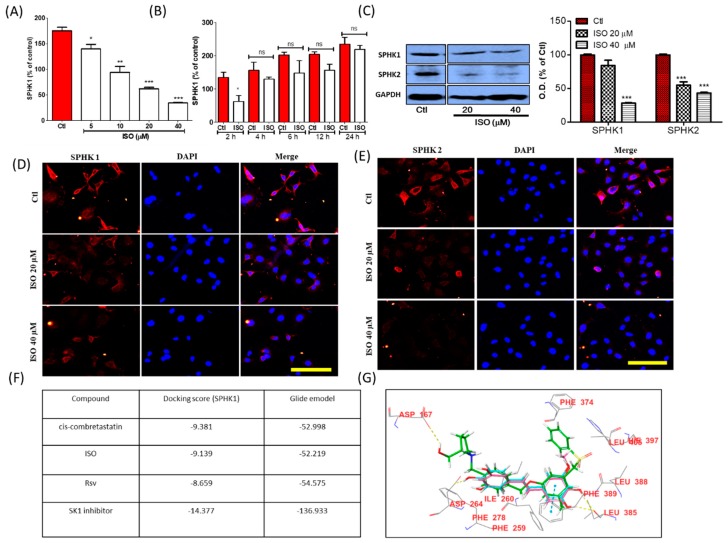
ISO treatment altered SPHK1/2 in MCF7 cells. (**A**,**B**) High throughput screening showing the concentration- and time-dependent inhibition of SPHK1 enzyme activity. MCF7 cells were treated with ISO for 48 h. (**C**) Protein expression of SPHK1 and SPHK2 and their densitometric analysis. GAPDH was used as a loading control. * *p* < 0.05, ** *p* < 0.01, and *** *p* < 0.001 indicate significant differences compared with untreated control group. (**D**,**E**) IF labeling of SPHK1/2, Scale bars: 50 µm. (**F**) SPHK1 docking score. (**G**) SPHK1 interaction with ISO (pink) and resveratrol (blue).

Sphingosine kinases (SPHK1/2) have recently become widely explored targets for cancer therapy. Very few phytochemicals or synthetic compounds are known to be regulators of sphingosine metabolism as well as SPHK modulators. Among these compounds, Rsv is a SPHK1 inhibitor that has been previously reported [9]. For the first time, we observed that ISO is also a great candidate for SPHK alteration in MCF7 cells. By means of high throughput screening, we observed that ISO treatment significantly inhibited SPHK1 enzyme activity in a concentration and time-dependent manner (Figure 2A,B). Inhibition of the protein expression of both SPHK1 and SPHK2 further confirmed the potency of ISO for modulating SPHKs in MCF7 cells (Figure 2C). Additionally, we performed IF/ICC for SPHK1 and SPHK2 and noted significant inhibition of SPHK1 and SPHK2 in MCF7 cells following ISO treatment (Figure 2D,E). We also confirmed the interaction of ISO with SPHK via a molecular docking study. As ISO showed greater ability to downregulate SPHK1, we targeted SPHK1 for the molecular docking study. We observed that ISO and Rsv both showed substantial interaction with SPHK1 (Figure 2F,G). Our results suggest that like Rsv, ISO can modulate SPHK1 expression and activity, and that ISO could be an excellent target for anti-cancer therapy against MCF7 cells.

### 3.3. ISO Treatment Induced ROS Production in MCF7 Cells

**Figure 3:**
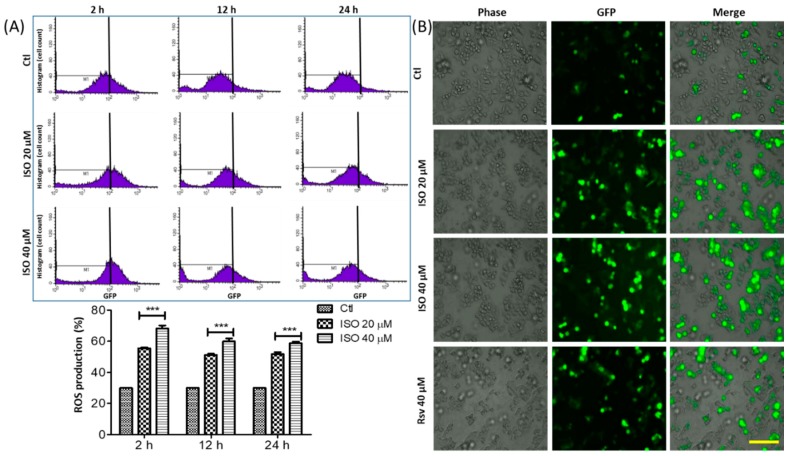
ISO treatment induced ROS production in MCF7 cells. MCF7 cells were treated with ISO for indicated time points. (**A**) FACS data showing the relative ROS production and their quantification. *** *p* < 0.001 indicates significant differences compared with untreated control group. (**B**) ROS production by MCF7 cells were evaluated by JULI imaging system using DCFDA-staining. Scale bar: 500 µm.

Reactive oxygen species (ROS) are key for generating oxidative stress and cellular damage. ISO treatment of MCF7 breast cancer cells significantly upregulated ROS production over three (2, 12, and 24 h) different periods of time (Figure 3). ROS production was higher over 2 h than 12 or 24 h, suggesting that ISO-mediated ROS upregulation occurs in the early stages of treatment. ISO treatment showed a clear increase in GFP-stained cells in a concentration-dependent manner in MCF7 cells (Figure 3). ISO-mediated ROS production in the early stages could generate huge oxidative stress and damage in MCF7 cells, leading to cell death/necrosis. 

### 3.4. ISO Induced Breast Cancer Cell Death through Caspase-Dependent Pathways

**Figure 4:**
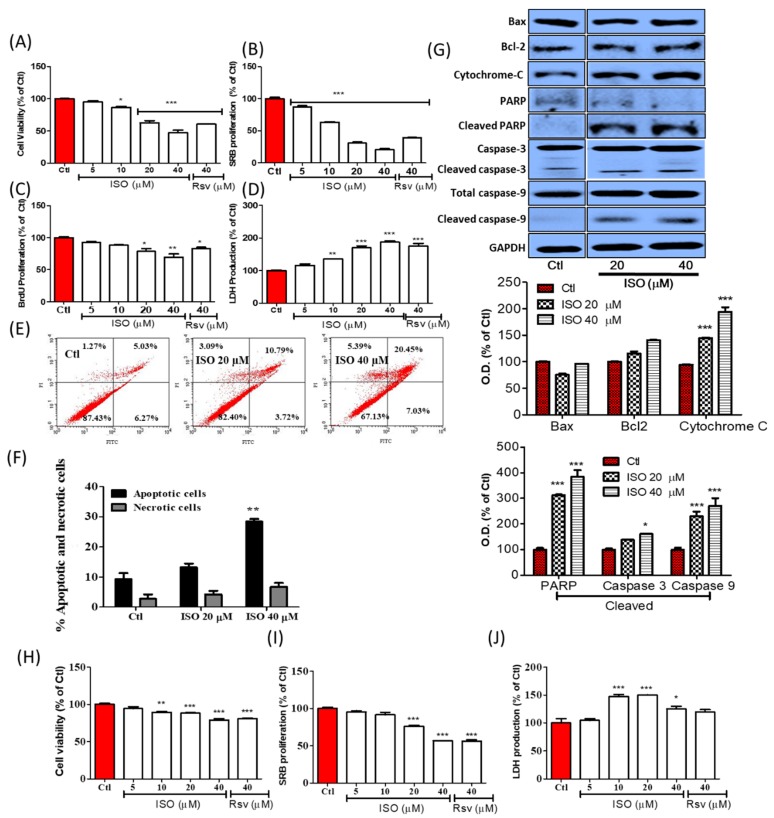
ISO treatment induced MCF7 cells (**A**–**G**) and T47D cells (**H**–**J**) death and growth arrest. Cells were treated with ISO and Rsv for 48 h. (**A**,**H**) Cell viability assay. (**B**,**I**) SRB proliferation assay. (**C**) BrdU proliferation assay. (**D**,**J**) LDH release assay. (**E**,**F**) Annexin V/PI apoptotic cell death assay. (**G**) Protein expression and their densitometric analysis of apoptosis-related proteins. Densitometric analysis of these proteins were calculated as cytosolic cytochrome C, cleaved PARP/PARP, cleaved caspase-3/caspase-3, cleaved caspase-9/caspase-9. GAPDH was used as a loading control. * *p* < 0.05, ** *p* < 0.01, and *** *p* < 0.001 indicate significant differences compared with untreated control group.

MCF7 breast cancer cells were treated with ISO and Rsv for 48 h. The cell viability and cytotoxicity of the treated samples were evaluated by MTT and LDH assays, while cell growth was evaluated by a sulforhodamine B (SRB) and BrdU proliferation assay. ISO treatment showed significant toxicity to MCF7 cells, starting from low concentrations. ISO showed almost 34.16 µM of IC50 for inducing MCF7 cell death. Treatment with 10–40 µM ISO significantly lowered cell viability and increased LDH production. Similarly, ISO treatment significantly lowered SRB and BrdU proliferation in a concentration-dependent manner. To compare the potency of ISO and Rsv treatment, treatment with these compounds was performed at a concentration of 40 µM. The results showed a clear difference, with ISO decreasing cell survival and proliferation (Figure 4A–D). Cell death/necrosis was further evaluated by annexin V/PI staining by flow cytometry. ISO treatment not only increased the number of early and late apoptotic cells, but also increased necrotic cells, suggesting that the treated concentration was sufficient for inducing significant cell death in MCF7 cells. Though both cell death events occurred in a concentration-dependent manner, apoptosis occurred to a higher extent than necrosis (Figure 4E,F). Despite the fact that, ISO treatment increased the number of both apoptotic and necrotic cells, there was no significant differences in necrotic cell death. This suggests that, necrosis induction might end with apoptosis hence ISO sharply increased the apoptotic cells and this could help for the inhibition of cancer growth. We also observed a significant increase in the expression of apoptosis-related proteins including cleaved PARP, cytoplasmic Cytochrome-C, cleaved caspase-3, and cleaved caspase-9, suggesting that apoptosis may be mediated in a caspase-dependent manner (Figure 4G). This was confirmed by the fact that ISO did not alter Bax and Bcl2 expression in MCF7 cells. ISO-mediated cell death was also analyzed by IncuCyte imaging, and the attached live cells were counted via Trypan blue staining and a live/dead cell assay (Figure S1). T47D cell is another type of breast cancer cell with similar characteristics with MCF7 [58], hence we confirmed the anti-cancer effect of ISO in T47D cells as well (Figure 4H–J). Treatment of ISO significantly reduced viable and proliferating T47D cells at 20 and 40 µM of concentration supporting our results for its effect in MCF7 cell. Additionally, ISO (10, 20, and 40 µM) significantly increased the level of LDH suggesting its role in inducing cellular toxicity. From these results, it can be hypothesized that ISO could induce strong anti-cancer effects in both MCF7 and T47D cells. 

### 3.5. ISO-Mediated SPHK1/2 Inhibition Is Responsible for Tubulin Destabilization, Cell Death, and Growth Arrest in MCF7 Cells

**Figure 5:**
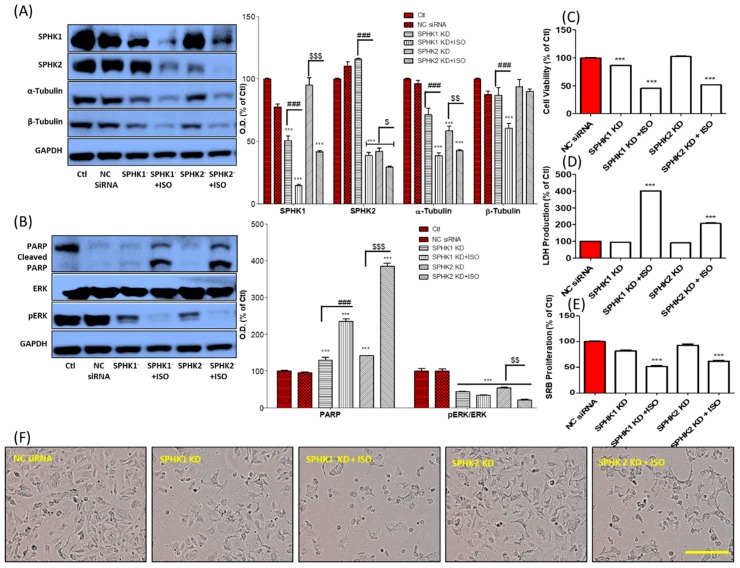
ISO-mediated SPHK1/2 inhibition was responsible for MCF7 cell death, growth arrest, and tubulin destabilization. SPHK1/2 gene silencing was performed for 6 h and ISO (40 µM) treatment was done for 48 h. (**A**,**B**) Protein expression of SPHK1/2, α/β-tubulin, cleaved PARP/PARP, pERK/ERK, and their densitometric analysis. GAPDH was used as a loading control. (**C**) Cell viability, (**D**) LDH production, and (**E**) SRB proliferation assay. *** *p* < 0.001 indicate significant differences compared with NC siRNA group while ^###^ *p* < 0.001 indicate significant differences compared with SPHK1 KD group. ^$^ *p* < 0.05, ^$$^ *p* < 0.01, and ^$$$^ *p* < 0.001 indicate significant differences compared with SPHK2 KD group. (**F**) Cell morphology. Scale bars: 500 µm.

To explore the role of ISO against MCF7 cells, particularly for tubulin depolymerization, we performed SPHK1/2 gene silencing/knockdown (KD) and compared cells treated with ISO to untreated controls. These results suggested that SPHK1/2 inhibition by ISO could be a key factor in the inhibition of α/β-tubulin expression, together with cleaved PARP activation and ERK phosphorylation inhibition (Figure 5A,B). SPHK1 KD showed great potency to lower the expression of α/β-tubulin while SPHK2 KD showed more specificity in lowering β-tubulin expression. SPHK1 KD and ISO treatment showed a clear inhibition of protein expression of α/β-tubulin, SPHK1/2, and pERK/ERK together with sharp increase of cleaved PARP. This result was further validated when we performed cell viability, LDH, and SRB assays under similar conditions (Figure 5C–E). SPHK1 inhibition-induced cell death, and the group with SPHK1 knockdown and ISO treatment showed the highest cell death, LDH production, and lowest cell proliferation. SPHK1/2 knockdown and ISO treatment dramatically increased cell death, growth arrest, and LDH production. These events can be observed in Figure 5F and Figure S2. Together, these results suggest that ISO-mediated SPHK1/2 inhibition could be responsible for tubulin destabilization, necrosis, and growth arrest of MCF7 cells. 

### 3.6. ISO-Mediated ROS Production and Anti-Inflammation in MCF7 Cells Could Be through SPHK1/SPHK2 Inhibition 

**Figure 6:**
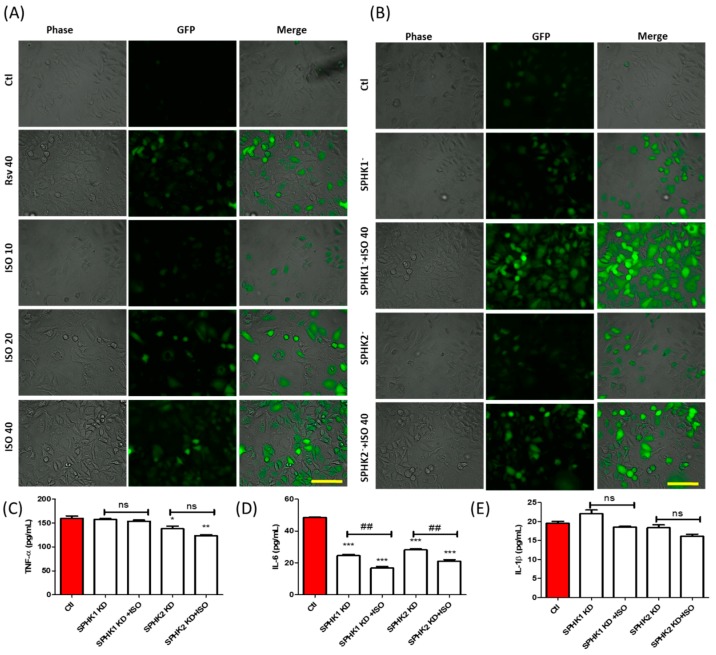
ISO-mediated SPHK1/2 inhibition was partly involved in ROS production and inhibition of pro-inflammatory cytokine production. SPHK1/2 gene silencing was performed for 6 h, and cells were treated with ISO for 48 h. (**A**,**B**) ROS production. Scale bars: 500 µm (**C**–**E**) Level of secreted pro-inflammatory cytokines, TNF-α, IL-6, and IL-1β. * *p* < 0.05, ** *p* < 0.01, and *** *p* < 0.001 indicate significant differences compared with untreated control group. ^##^ *p* < 0.01 indicate significant differences compared with SPHK KD group, ns: not significant.

After confirming the role of SPHK1/2 in tubulin polymerization, cell death, and proliferation, we investigated its effect on ROS and pro-inflammatory cytokine production. MCF7 cells were treated with different concentrations of ISO and one high dose of Rsv. At the same time, SPHK1/2 knockdown and ISO co-treatment was performed, and ROS production was measured. ROS production increased in a manner dependent on ISO concentration. ISO-mediated ROS production was much higher at 40 µM ISO than Rsv treatment alone, as shown in Figure 6A. SPHK1 and SPHK2 knockdown (KD) significantly increased ROS production. This was further accelerated by co-treatment with ISO, suggesting that in the ROS production cascade, SPHK1/2 could actively participate in the induction of oxidative stress (Figure 6B). In addition to this, SPHK1 KD significantly inhibited IL-6 production, and this was further accelerated by ISO treatment. This suggested that ISO-mediated IL-6 production could possibly be mediated by SPHK1 inhibition. SPHK2 KD inhibited TNF-α and IL-6 production, and both were more reduced in the SPHK2 KD and ISO co-treatment groups. SPHK1 and SPHK2 KD did not alter IL-1β production, indicating that ISO-mediated SPHK1/2 inhibition may not be responsible for IL-1β inhibition, but may instead occur through other pathways. Though ISO co-treatment slightly decreased IL-1β production, this was not significant (Figure 6C–E).

### 3.7. ISO Inhibited Phosphorylation of the MAPK/Pi3K Signaling Pathway and Pro-Inflammatory Cytokine Production in MCF7 Cells 

**Figure 7:**
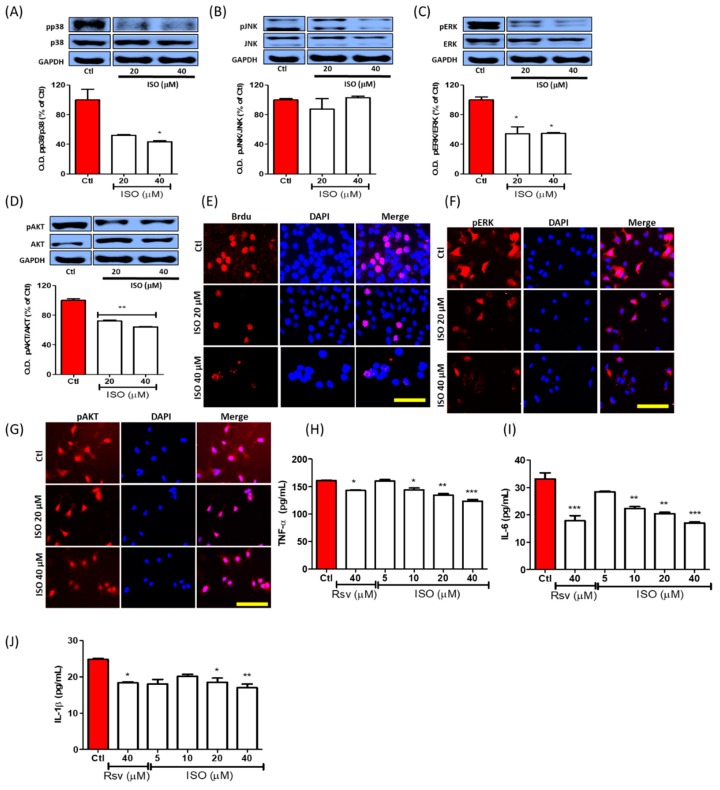
ISO treatment controlled MAPK/PI3K signaling and downregulated cell proliferation and pro-inflammatory cytokine production. MCF7 cells were treated with ISO and Rsv for 3 h for MAPKs and PI3K, and 48 h for cytokines assay. (**A**–**D**) Protein expression of MAPKs and PI3K/AKT and their densitometric analysis. (**E**) IF labeling of BrdU-positive proliferating cells. (**F**,**G**) IF labeling of pERK and pAKT. Scale bars: 50 µM (**H**–**J**) Level of secreted pro-inflammatory cytokines, TNF-α, IL-6, and IL-1β. * *p* < 0.05, ** *p* < 0.01, and *** *p* < 0.001 indicate significant differences compared with untreated control group.

MCF7 breast cancer cells were incubated with ISO for 3 h. ISO treatment significantly inhibited the phosphorylation of p38, pERK, and pAKT. Inhibition of pERK and pAKT activation is directly linked with the inhibition of cell proliferation (Figure 7A–D). As previously demonstrated, ISO inhibited breast cancer cell proliferation, and this could possibly be due to inhibition of ERK and AKT phosphorylation. BrdU staining further supported the idea that cell proliferation is regulated by ISO treatment (Figure 7E). In addition to this, conducting immunofluorescence analysis of the MCF7 cells for pERK and pAKT clearly supported the results observed via Western blot analysis (Figure 7F,G). These results indicated more noticeable pERK inhibition in both analyses, suggesting that proliferation inhibition by ISO could be majorly regulated by ERK, in addition to being supported by AKT inhibition. As inhibition of MAPK signaling is well reported to have strong anti-inflammatory effects, this could explain the strong inhibition of the expression of pro-inflammatory cytokines such as TNF-α, IL-6, and IL-1β in MCF7 cells following ISO treatment (Figure 7H–J). Non-specific pro-inflammatory cytokine inhibition by ISO treatment further expands on its biological potency for decreasing cancer-mediated inflammation, particularly in MCF7 cells.

### 3.8. ISO-Mediated Anti-Proliferation Was further Increased by Co-Treatment with ERK and AKT Inhibitor

**Figure 8:**
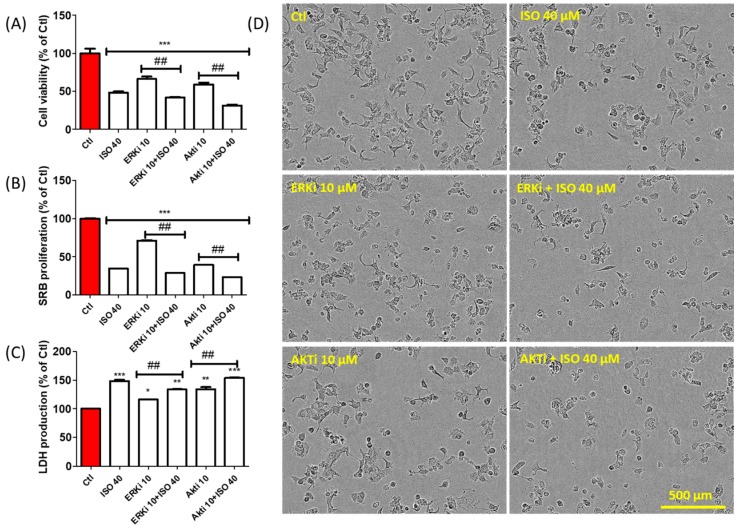
ERK and AKT inhibitor treatment accelerate the anti-cancer effect of ISO in MCF7 cells. MCF7 cells were treated with ISO and EKR inhibitor and AKT inhibitor for 48 h. (**A**) Cell viability assay. (**B**) SRB proliferation assay. (**C**) LDH production assay. * *p* < 0.05, ** *p* < 0.01, and *** *p* < 0.001 indicate significant differences compared with untreated control group. ^##^ *p* < 0.01 indicates significant differences compared with ISO (40 µM) treated group. (**D**) Cell morphology.

ISO treatment decreased breast cancer cell proliferation *via* downregulation of ERK and AKT signaling, followed by G2/M phase cell cycle arrest. This result was further validated by the observation that cell death and proliferation inhibition were increased by co-treatment of ISO with the ERK and AKT inhibitors U0126 and LY294002, respectively (Figure 8A–C). This can be observed further in Figure 8D. These results suggest that ISO inhibits breast cancer cell growth and proliferation via inhibition of ERK/AKT phosphorylation. This is followed by G2/M cell cycle arrest, which is mostly responsible for tumor cell proliferation. 

### 3.9. ISO Regulates G0/G1 and G2/M Phase Arrest in MCF7 Cells

**Figure 9:**
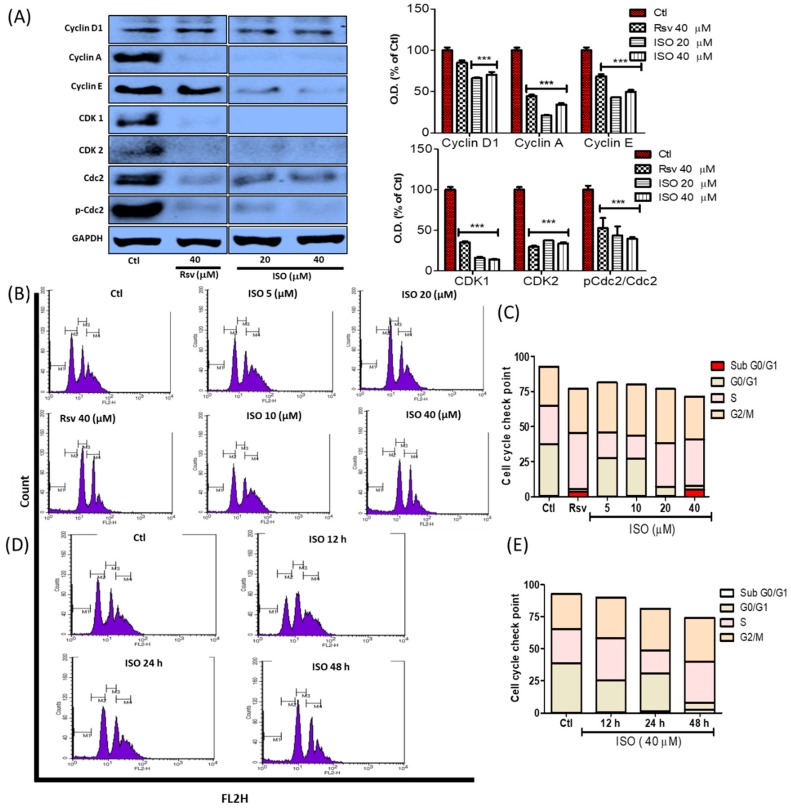
ISO treatment induced cell cycle checkpoint arrest in MCF7 cells. MCF7 cells were treated with ISO and Rsv for 48 h. (**A**) Protein expression of several cell cycle checkpoint-related proteins and their densitometric analysis. GAPDH was used as a loading control. *** *p* < 0.001 indicate significant differences compared with untreated control group. (**B**,**C**) Cell cycle differentiation assay in a concentration-dependent study. (**D**,**E**) Cell cycle differentiation assay in a time-dependent study.

Cell death or cell growth arrest by inhibiting cell proliferation occurs through cell cycle arrest. Throughout this process, several cell cycle proteins become altered following treatment, ultimately leading to cell growth or death depending on the biological activity of the treatment compounds. As ISO treatment led to G0/G1 and G2/M phase arrest via increased cell death and reduced cell proliferation, we further investigated the expression of cell cycle checkpoint proteins. Inhibition of the G0/G1 and G2/M phase is mostly correlated with the decrease of several cyclin proteins. We observed that ISO treatment dramatically reduced cyclin D, cyclin E, and cyclin A expression. In addition to this, clear inhibition of CDK 1, CDK 2, and activated cdc2 was observed. Though this alteration in protein expression was also induced by Rsv treatment, ISO demonstrated this more effectively (Figure 9A). Apoptosis induction was observed through G0 arrest. Furthermore, increased cell accumulation in the G2/M phase suggested that the ISO-mediated anti-cancer effect occurs through G2/M phase arrest. Through this phase arrest, cancer proliferation was altered or reduced. In both the time and concentration-dependent studies, G0/G1 and G2/M phase arrest was observed, revealing that G0/G1 and G2/M phase arrest occurs due to ISO treatment in breast cancer cells (Figure 9B–E). 

### 3.10. ISO-Mediated Anti-Cancer Effect Was Recovered by Sirt1 Inhibitor Nicotinamide

**Figure 10:**
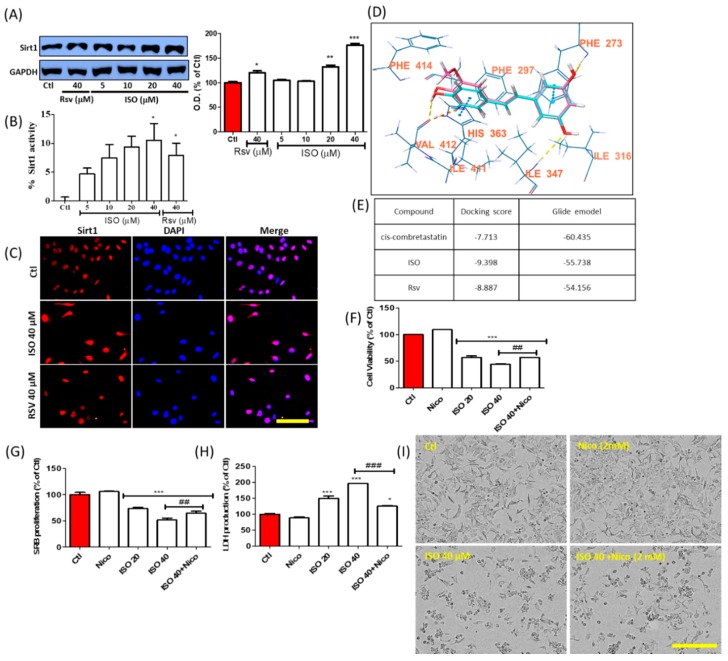
ISO treatment activated Sirt1 in MCF7 cells. MCF7 cells were treated with ISO and Rsv for 48 h. (**A**) Protein expression of Sirt1 and densitometric analysis. GAPDH was used as a loading control. (**B**) Sirt1 activity assay. (**C**) IF labeling of Sirt1. (**D**) Sirt1 interaction with isorhapontigenin (pink) and resveratrol (blue). (**E**) Sirt1 docking score. (**F**) Cell viability assay. (**G**) SRB proliferation assay. (**H**) LDH release assay. (**I**) Cell morphology. Scale bars: 50 µm in C and 500 µm in I. * *p* < 0.05, ** *p* < 0.01, and *** *p* < 0.001 indicate significant differences compared with untreated control group. ## *p* < 0.01, and ### *p* < 0.001 indicate significant differences compared with ISO 40 µM-treated group.

ISO being an analog of resveratrol, we evaluated its role in expression and activity of Sirt1. Treatment of Rsv and ISO significantly increased the Sirt1 expression (Figure 10A) where ISO showed better potency. Induction of Sirt1 activity (Figure 10B) and intracellular expression (Figure 10C) by ISO in Sirt1 activity assay and IF assay further confirms the Sirt1 activating potential of ISO. As expected, ISO showed better docking score and binding affinity (Figure 10D,E) to Sirt1 in comparison to Rsv itself in the molecular docking study. Altogether, these results suggested the Sirt1 activating potential of ISO. To evaluate the role of Sirt1 that has been activated by ISO in MCF7, we performed the experiment using Sirt1 inhibitor. MCF7 cells were treated with ISO alone and with ISO together with the Sirt1 inhibitor nicotinamide. Co-treatment of ISO with nicotinamide significantly recovered cells from death, proliferation, and LDH production (Figure 10F–H). Representative pictures of the treatment also clearly show the differences between treatment with ISO alone and with ISO and nicotinamide (Figure 10I). This result suggests that ISO-mediated cell damage/death and proliferation is mediated through Sirt1 activation. 

**Figure 11:**
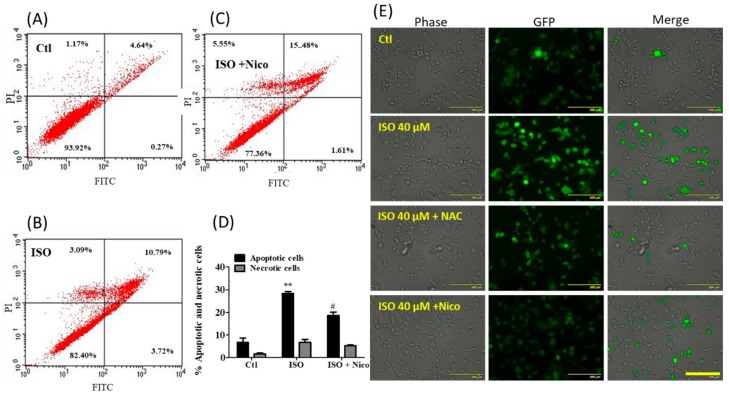
Nicotinamide treatment opposed the anti-cancer effect of ISO in MCF7 cells. MCF7 cells were treated with ISO (40 µM) and nicotinamide (2 mM) for 48 h. (**A**–**D**) Cell apoptosis assay using annexin V/PI staining. ** *p* < 0.01 indicates significant differences compared with the untreated control group, while ^#^ *p* < 0.05 indicates significant differences compared with only ISO treated group. (**E**) ROS production measurement by DCF-DA staining, photographed by using JULI imaging system, Scale bar: 500 µm.

To confirm the effect of ISO through Sirt1, we further validated our results by performing annexin V/PI staining. ISO treatment significantly induced cell apoptosis and necrosis, and both of these were recovered by co-treatment with nicotinamide (Figure 11A–D). The same pattern was noted for ROS production (Figure 11E). Altogether, these results suggest that Sirt1 might partially involve in the ISO-mediated cell death and ROS production in MCF7 cells. 

### 3.11. ISO Induced MDA-MB-231 Cell Death and Growth Arrest

**Figure 12:**
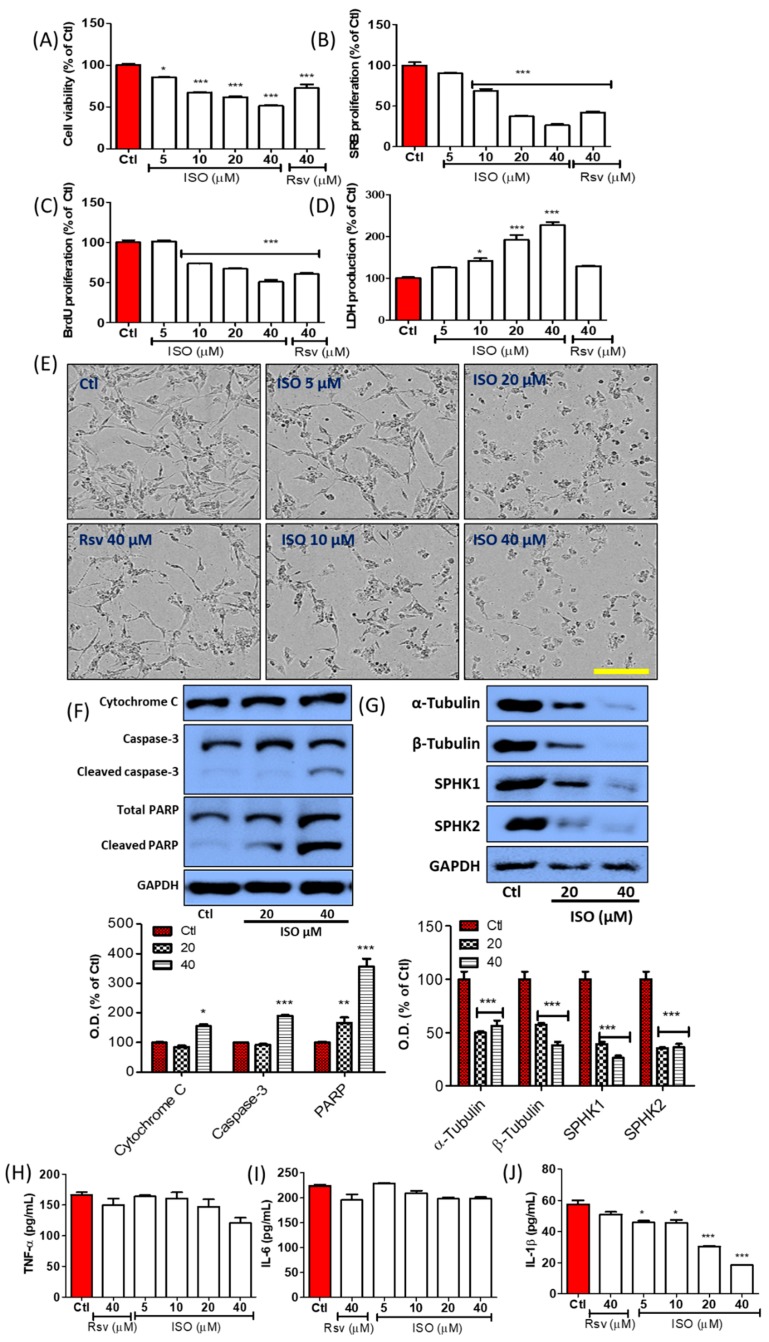

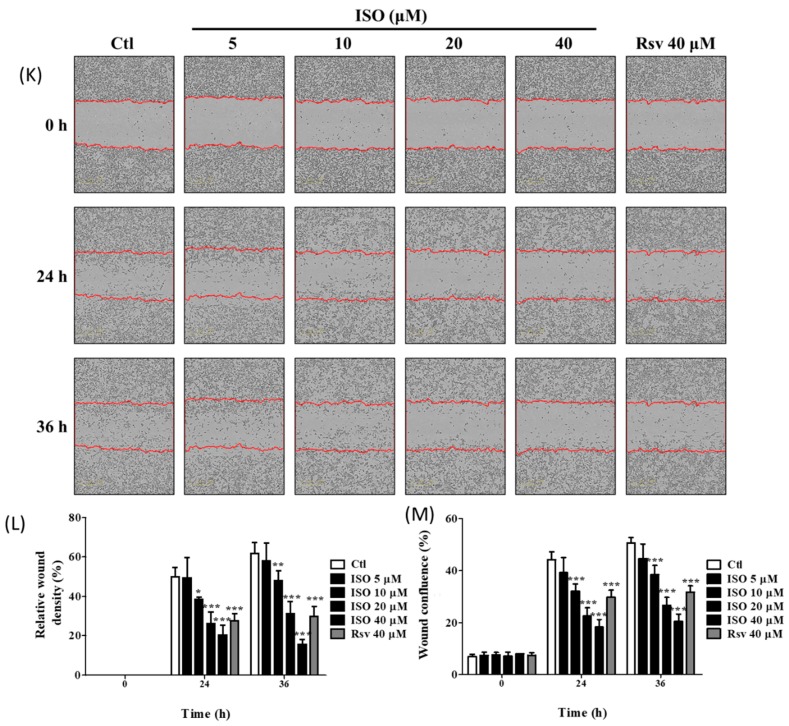
ISO treatment increased cell death, growth arrest, and attenuated migration in MDA-MB- 231 cells. MDA-MB-231 cells were treated with ISO. (**A**) Cell viability assay. (**B**) SRB proliferation assay. (**C**) BrdU proliferation assay. (**D**) LDH production assay. (**E**) Cell morphology, scale bar: 500 µM. (**F**,**G**) Protein expression of cytochrome C, PARP, caspase-3, SPHK1, 2, and α, β tubulin. Densitometric analysis of these proteins was calculated as cytoplasmic cytochrome C, cleaved caspase-3/caspase-3, cleaved PARP/PARP, SPHK1, 2/GAPDH, and tubulin/GAPDH. GAPDH was used as a loading control. (**H**–**J**) Secreted level of TNF-α, IL-6, IL-1β. (**K**) Cell migration assay using the IncuCyte imaging system for wound healing. (**L**) Relative wound density. (**M**) Wound confluence (%). * *p* < 0.05, ** *p* < 0.01, *** *p* < 0.001 indicate significant differences compared with the untreated control group.

Anti-cancer effect of ISO against breast cancer was further confirmed in triple-negative breast cancer cell line (MDA-MB-231-231). Like in MCF7 and T47D cells, ISO treatment induced cell death and growth arrest in MDA-MB-231 cells as well. As evidenced by MTT assay, ISO treatment concentration-dependently reduced the number of viable cells suggesting induced cell death upon ISO exposure (Figure 12A). ISO showed better potency than Rsv to induce MDA-MB-231 cell death. Not only this, ISO and Rsv treatment lowered the MDA-MB-231 cell proliferation (Figure 12B,C), suggesting its role in the growth arrest. Increased LDH production by damaged cells following ISO treatment reveled the potency of ISO on inducing cytotoxicity and oxidative stress TNBCs (Figure 12D). Cell death and morphological changes were observed by IncuCyte imaging (Figure 12E). As an underlined mechanism, ISO induced the level of cytoplasmic cytochrome C, cleaved caspase-3, and cleaved PARP (Figure 12F). ISO treatment showed a clear inhibition of SPHK1/2 and tubulin in MDA-MB-231 cells as well, suggesting that inhibition of the SPHKs and tubulin polymerization could be the responsible pathways for the ISO mediated TNBCs death and growth arrest (Figure 12G). Altogether, our results suggest the strong potential of ISO for the anti-cancer effect against TNBCs as well. These effects are similar to that of MCF7 suggesting a similar mechanism of ISO in both of these breast cancer cells. In addition to this, ISO treatment did not alter the level of secreted TNF-α and IL-6; however, treatment of ISO concentration-dependently inhibited the IL-1β production, suggesting that ISO mediated anti-inflammatory effect against TNBCs might be regulated through IL-1β inhibition (Figure 12H–J). In spite of being breast cancer cells, TNBCs and non-TNBCs have different characteristics of cell growth. TNBCs (MDA-MB-231) are more aggressive than non-TNBCs like MCF7/T47D cells. Hence we evaluated the role of ISO in controlling the MDA-MB-231 cell migration, and this experiment was performed by scratch wound assay. As ISO inhibited SPHK1/2 in MDA-MB-231 cells, it significantly lowered its migration as well, and it was quantified by relative wound density and relative wound confluence (Figure 12K–M). ISO showed higher potency to lower MDA-MB-231 cell migration in comparison to Rsv at the same concentration.

## 4. Discussion

Rsv and its derivatives are well reported to have strong anti-cancer effects against several cancer types. However, ISO is a Rsv derivative that has not been thoroughly reported on. Except for in bladder cancer, ISO could potentially have similar or different anti-cancer effects through similar or differential targets than Rsv. Rsv activates Sirt1, promotes tubulin destabilization and inhibits SPHK, thus making it a potential phytochemical for treatment strategies against breast cancer. However, issues with its bioavailability and stability limit its effectiveness [7,26,59,60,61]. Rsv-mediated SPHK1 inhibition has been shown to induce K562 leukemia cancer cell death [60]. Rsv-mediated tubulin destabilization has also been observed to induce mitotic arrest and cancer cell death via G2/M phase arrest [26]. Sirt1 activation by Rsv also induces human chondrosarcoma cell apoptosis through intrinsic apoptotic pathways [59]. In this study, we revealed the higher potency of ISO against breast cancer by inducing cell death and growth arrest through similar targets. As ISO is a more readily bioavailable polyphenol and analog of Rsv, this compound could counter the limitations of Rsv and act as an alternative for possible treatment against breast cancer. In this study, we investigated the strong anti-cancer effects of ISO, which is almost 2-fold higher than Rsv, against breast cancer cell survival and growth. The anti-cancer effects of ISO were strongly correlated with the activation of apoptotic pathways, tubulin destabilization, SPHK1/2 inhibition, and Sirt1 activation. 

Alteration in tubulin polymerization/stabilization was reported to be involved in cell cycle arrest, apoptosis, cell growth inhibition, and cell damage [62]. Interestingly, ISO treatment showed clear inhibition of polymerized tubulin expression, suggesting the possible involvement of ISO in tubulin polymerization. Tubulin destabilizing ability was also confirmed by IF/ICC staining. It can be seen from our results that ISO and Rsv have high structural similarities with combretastatin, a colchicine analog known to interact with the colchicine-binding site in tubulin [63,64]. Rsv is well known to interact with tubulin, and therefore ISO may also do so by maintaining its structural similarity with Rsv [26,57,65]. The docking results reconfirmed that ISO and Rsv bind to the colchicine binding site in the same orientation as combretastatin, and with a similar docking score. ISO and Rsv are highly similar to the cis-combretastatin structure, thus resembling the interaction. Rsv showed a high affinity towards tubulin but had a lower affinity than that of ISO and cis-combretastatin. Though the SPHK1 inhibitor did not show a high affinity towards tubulin, direct protein–protein interaction could be possible via several indirect pathways. Sphingosine modulators were also reported to be linked with tubulin disruption and have subsequent anti-cancer effects. Previous reports have suggested that SKI-178, being a sphingosine kinase inhibitor, functioned as a microtubule network-disrupting agent, showing strong anti-cancer potency by inducing acute myeloid leukemia cell death [24]. ISO dramatically lowered the expression and activity of SPHKs. Additionally, docking studies with SK1 protein were conducted to validate the interaction of ISO and Rsv with SK1 [24,66]. The docking results suggest that both compounds have the potential to interact with SK1 protein, but their binding potential is weaker than that of the known SK1 inhibitor. Moreover, ISO demonstrated a stronger interaction than Rsv. This strong ability of ISO to lower SPHKs suggests the possibility of it lowering cancer cell resistance, thus increasing the sensitivity of cells to therapy. This is because AKT, SPHK1, and SPHK2 are reported to be involved in cancer cell growth and resistance. These proteins are key prognostic factors for several cancer types, including breast cancer [16,17,18,19,22,23]. Furthermore, treatment of chemo and hormone therapy-resistant breast cancer with the SPHK2 inhibitor ABC294640 completely inhibited tumor volume, suggesting that pharmacological inhibition of SPHK2 could be a strong anti-cancer strategy [20,67]. As SPHK inhibitors like Rsv are also tubulin destabilizers, these show strong anti-cancer effects. Similarly, tubulin destabilizers or stabilizers like vinca and paclitaxel can affect microtubule dynamics, hence lowering the survival and growth of cancer cells [25,62]. Moreover, inhibition of SPHK1/2 and induction of tubulin destabilization by ISO greatly influenced breast cancer cell death and growth. This further insinuates that ISO and these targets are key for developing better strategies for breast cancer therapy. Additionally, ISO-mediated ROS production could also participate in the upregulation of oxidative stress, and this could be responsible for cancer cell death and growth arrest. This notion was supported by ISO treatment inducing the apoptosis, necrosis, and proliferation reduction of MCF7 cells, suggesting strong anti-cancer effects against breast cancers. Although a strong increase in P53, cytoplasmic Cytochrome-C, cleaved caspase-9, cleaved caspase-3, and cleaved PARP expression was observed, no significant alteration in Bax and Bcl2 levels was observed. This result suggested that ISO-mediated breast cancer cell death could occur through mitochondria-mediated caspase-dependent pathways. This was supported by a previous finding in which Rsv showed similar effects, inducing breast cancer cell death [68,69]. Similarly, Wu et al. also revealed that increased P53 and the release of mitochondrial cytochrome C to the cytoplasm leads to cellular oxidative stress, resulting in the activation of caspases through caspase cleavage [70]. An increase in cleaved caspase-8/9 further upregulated caspase-3 cleavage, leading to cleavage/activation of PARP, which then blocked DNA repair. Such cascades ultimately caused oxidative stress, cell necrosis, or apoptosis [70]. Previous reports suggested that T47D cells are hormone-dependent breast cancer cells having similar characteristics to that of MCF7. Hence, we determined the anti-cancer effects of ISO in T47D cells as well. As expected, ISO treatment significantly increased cell death, LDH production while it suppressed cell proliferation of T47D cells.

The strong ability of ISO to inhibit MCF7 cell survival and growth, inhibit SPHK, and induce tubulin destabilization suggested a relationship between SPHK, tubulin, and the fate of MCF7 cells. Both SPHK1/2 alone and SPHK1/2 KD together with ISO treatment strongly reduced the expression of α/β-tubulin and pERK, in addition to increasing cleaved PARP. A previous study also revealed similar results, in which a multitargeted SPHK inhibitor participated in microtubule dynamic disturbances [24]. As these target genes are critically involved in the proliferation and survival of cancer cells, alteration of these genes by SPHK1/2 could be responsible for anti-cancer activity. This was confirmed in the ISO-treated group with SPHK1/2 KD [71,72,73]. SPHK inhibition, either genetically or pharmacologically, inhibited cell viability and proliferation, followed by the induction of LDH and ROS production. These results are supported by findings obtained by Hamada et al., who reported that SPHK1/2 is involved with cell death, proliferation, ROS production [74]. Furthermore, we observed that SPHKs are involved in inhibiting cancer-mediated inflammation. More specifically, SPHK1/2 KD is involved in the inhibition of IL-6 production in MCF7 cells, thus reducing cancer-mediated inflammation. Our data correlate with a previous finding in which SPHK1 inhibition lowered the production of inflammatory mediators such as TNF-α, IL-6, IL-1β, whereas SPHK2 inhibition lowered the production of TNF-α and IL-1β in macrophages [75,76]. Overall, SPHK1/2 are inflammatory, and hence inhibition of SPHK1/2 decreases cancer-mediated inflammation. A similar result was observed here, and SPHK1/2 inhibition by ISO controlled IL-6 production, further decreasing the activity of inflammatory cascades.

Cell survival or death and inflammation induction in cancers are mostly mediated by mitogen-activated protein kinase (MAPK) and PI3K signaling [77]. Among these, ERK and AKT phosphorylation highly focus on cancer cell survival and growth/proliferation [78,79]. This correlates with our results, wherein ISO treatment reduced the activation of all MAPK and PI3K signaling. MAPK inhibition by ISO treatment resulted in decreased pro-inflammatory cytokine production. Furthermore, inhibition of ERK and AKT activation was also possibly involved in MCF7 cell death and reduced cell proliferation. As ERK and AKT are the major signaling pathways that participate in MCF7 cell death and growth inhibition by ISO, pharmacological inhibition of this signaling using their respective inhibitors revealed their exact involvement in ISO-mediated MCF7 cell death and growth inhibition.

Cancer cell death and growth arrest are mechanistically mediated by cell cycle checkpoint arrest [80]. This was evidenced by our results, wherein ISO treatment increased G0/G1 and G2/M phase arrest. G0/G1 phase arrest resulted in cell death, and the G2/M phase arrest resulted in the inhibition of cancer cell growth through reduced cell proliferation [81,82]. Upon comparing this with our results, it was summarized that ISO-mediated G0/G1 arrest could be responsible for the induction of mitochondria-mediated caspase-dependent breast cancer cell death, as discussed earlier, and that G2/M phase arrest could be responsible for reduced proliferation. These results were further supported by the reduced expression of cell cycle checkpoint proteins. ISO treatment dramatically reduced the expression of tubulin and checkpoint proteins like Cyclin (A, D, E), CDK (1, 2), and activated cdc2. Interestingly, G1 and G2 cell cycle inhibition is known to be regulated by microtubule depolymerization in human breast cancer, suggesting that ISO-mediated G0/G1 and G2/M phase arrest may occur through its ability to induce microtubule depolymerization [83].

As Rsv is a potent Sirt1 activator and ISO is an analog of Rsv, it is logical to infer the possible effect of ISO on Sirt1 activation [84]. As expected, ISO treatment not only increased the expression and activity of Sirt1 more effectively than Rsv, it also showed higher Sirt1 binding ability in the molecular docking experiment. This suggested that the potency of ISO to upregulate Sirt1 is better than that of Rsv. The involvement of Sirt1 activation in ISO treatment was confirmed by our results, wherein ISO co-treatment with the Sirt1 inhibitor nicotinamide significantly reduced the effect of ISO against MCF7 cells, either by increasing cell survival or proliferation or by decreasing LDH release. Nicotinamide and N-acetyl cysteine treatment reduced ISO-mediated ROS production.

Rsv was reported to have anti-cancer efficacy for both TNBC and Non-TNBCs [85]. In this study, ISO showed efficacy to induce the non-TNBC cell death and growth arrest; we expected a similar effect of ISO in TNBCs as well. As expected, ISO treatment showed a sharp decline in cell viability and proliferation of MDA-MB-231 cells. Increased LDH release, apoptotic protein expression, and altered cellular morphology by ISO exposure suggested the apoptosis- and necrosis-mediated damage of MDA-MB-231 cells following ISO treatment. Additionally, ISO treatment lowered the expression of SPHK1/2 and polymerized tubulin supporting the fact that inhibition of the SPHKs and tubulin polymerization could be a reason behind the anti-cancer effect of ISO against TNBC and non-TNBC cell lines. Interestingly, ISO-treatment significantly lowered the level of IL-1β but not TNF-α and IL-6 in MDA-MB-231 cells. However, ISO treatment significantly downregulated the levels of all these cytokines in MCF7 cells. Although this disparity between these cells for TNF-α was not reported previously, the difference in IL-6 expression was previously reported [86]. Inconsistent with our findings, ISO treatment lowered the level of IL-6 only in MCF7. TNBC cells (MDA-MB-231) express a higher level of pro-inflammatory cytokines than that of non-TNBC cells (MCF7). This could be a reason that ISO treatment inhibited IL-6 production in MCF7 but not in MDA-MB-231 cells. In addition, ISO-mediated specific inhibition of the secreted level of IL-1β suggested its anti-inflammatory effects, which could be of interest for further investigation about the anti-cancer and anti-inflammatory effects of ISO. ISO-mediated inhibition of the MDA-MB-231 cell migration further supports the anti-cancer effect of ISO against TNBC cells. Further studies on the effect of ISO against TNBCs are needed. 

## 5. Conclusions

ISO treatment showed a higher potency for inducing MCF7, T47D, and MDA-MB-231 cell death and growth arrest through activation of caspase-dependent cell death cascades. ISO-mediated downregulation of SPHK1/2 and increased tubulin destabilization can be the major targets involved in the strong anti-cancer effect of ISO against breast cancers. This can be applied for inducing cancer cell death and growth arrest in both TNBC and non-TNBC cell lines. Validation of this experiment through in vivo research and further advanced studies could establish ISO as a better option than Rsv for the treatment of several cancer types, particularly breast cancer.

## Supplementary Materials

The following are available online at https://www.mdpi.com/2072-6694/11/12/1947/s1, Figure S1: ISO treatment inhibited the number of viable cells and increased the dead ones, Figure S2: SPHK1/2 inhibition by gene silencing or through ISO treatment induced MCF7 cell death.
