# Supplementary material for: A Stilbenoid Isorhapontigenin as a Potential Anti-Cancer Agent against Breast Cancer through Inhibiting Sphingosine Kinases/Tubulin Stabilization
Source: Cancers (Basel). 2019 Dec 5;11(12):1947. doi: 10.3390/cancers11121947 (PMC6966567; doi:10.3390/cancers11121947)
Supplement: Supplementary file 1 [file cancers-11-01947-s001.zip › cancers-643215-supplement--.pdf]

Methods

1. Live/dead cell assay

Cellular toxicity by ISO was measured via a live/dead assay kit. This kit stained live cells with green fluorescence and dead cells with red fluorescence due to the presence of calcein-AM and ethidium homodimer-1 (EthD-1), respectively. In brief, MCF-7 and MDA-MB 231 cells were seeded and treated with the appropriate compound for approximately 48 h. After treatment, 2 μM calcein-AM and 4 μM EthD-1 working solution was added to the conditioned medium and incubated for 30 min. The green and red fluorescence of the live and dead cells was photographed with IncuCyte imaging system. This experiment was performed according to the protocol mentioned in the kit manual.

2. Trypan blue staining

To confirm the cell death with the treatment of Isorhapontigenin, Trypan blue staining was performed. Cells were treated for 48 h with ISO and Rsv. Treated cells were washed with PBS and Trypsinized to detach the cells. Trypsinized cells were suspended with Equal volume of 0.05 % Trypan blue and medium and let them stained for some time in order to let the viable and nonviable cells to stain with Trypan blue. Stained cells were counted using haemocytometer. In this counting process the viable cells showed a clear cytoplasm while the dead cells presented blue cytoplasm.

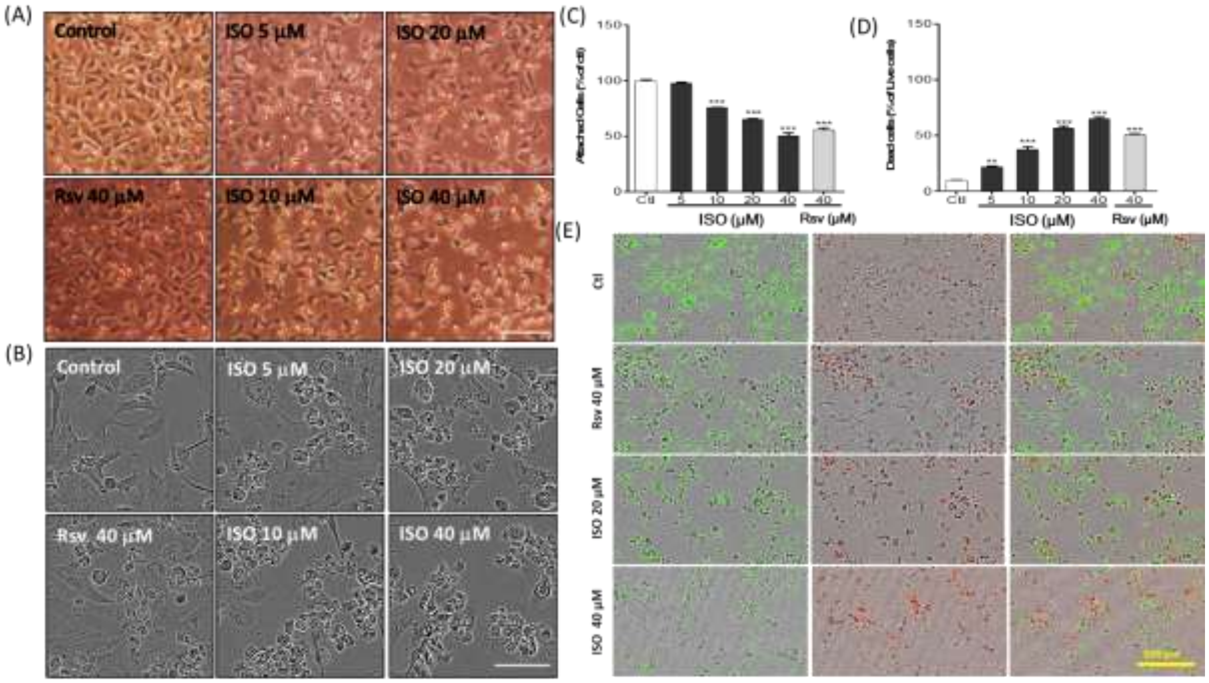

Supplementary figure 1. ISO treatment inhibited the number of viable cells and increased the dead ones. MCF7 cells were treated with ISO (5, 10, 20, 40 μM) and Rsv (40 μM) for 48 h. (A) Images were captured at the end of treatment by using 10X microscopic camera. (B) Images were captured by using IncuCyte ZOOM (Essen Bioscience). (C) Attached cells were evaluated by using IncuCyte ZOOM live cell imaging and analyzing tool. (E) Dead cells were determined by using Trypan blue staining. **p < 0.01 and ***p < 0.001 indicate significant differences compared with untreated control group. (E) Live/dead cells were stained by using live/dead cells staining assay.

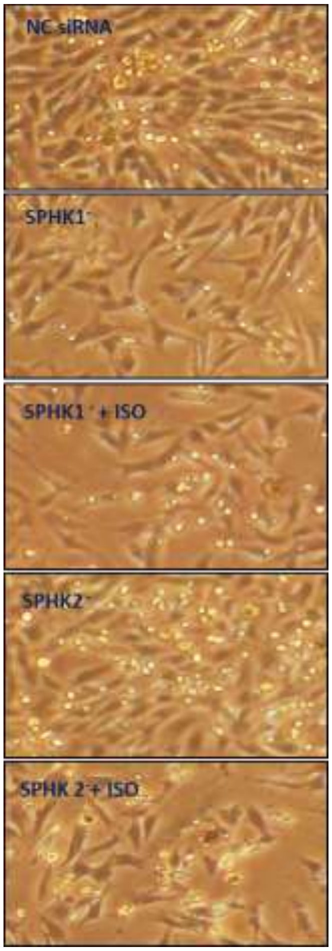

Supplementary figure 2. SPHK1/2 inhibition by gene silencing or through ISO treatment induced MCF7 cell death.
