# Supplementary material for: A Stilbenoid Isorhapontigenin as a Potential Anti-Cancer Agent against Breast Cancer through Inhibiting Sphingosine Kinases/Tubulin Stabilization
Source: Cancers (Basel). 2019 Dec 5;11(12):1947. doi: 10.3390/cancers11121947 (PMC6966567; doi:10.3390/cancers11121947)
Supplement: Supplementary file 1 [file cancers-11-01947-s001.zip › Original Western blot data.pdf]

[Cancers] Manuscript ID: cancers-643215

Title: A stilbenoid Isorhapontigenin as a potential anticancer agent against breast cancer through inhibiting sphingosine kinases/tubulin stabilization

Authors: Lalita Subedi, Mahesh Kumar Teli, Jae Hyuk Lee, Bhakta Prasad Gaire, Mi-hyun Kim, Sun Yeou Kim

Original Western blot

Figure 1 original data

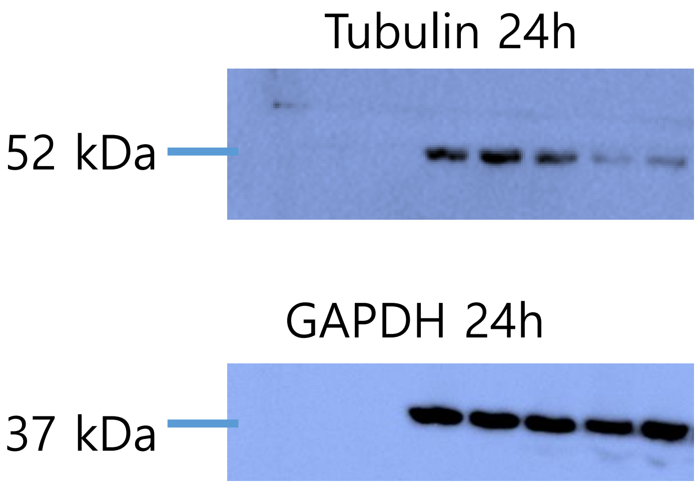

Band 1: Ctl
Band 2: ISO 5 μM
Band 3: ISO 10 μM
Band 4: ISO 20 μM
Band 5: ISO 40 μM

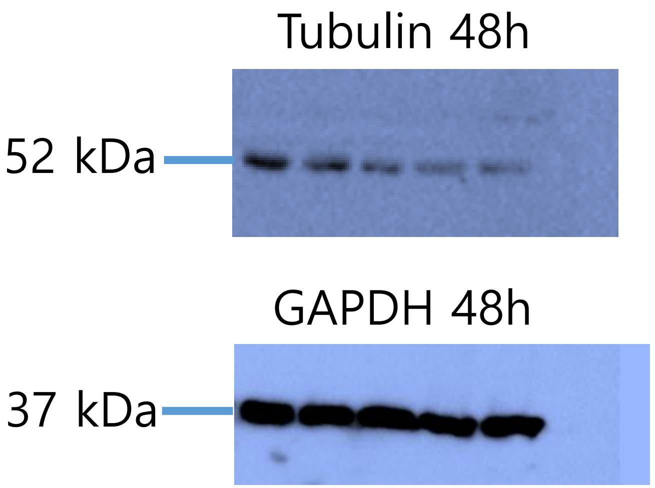

Band 1: Ctl
Band 2: ISO 5 μM
Band 3: ISO 10 μM
Band 4: ISO 20 μM
Band 5: ISO 40 μM

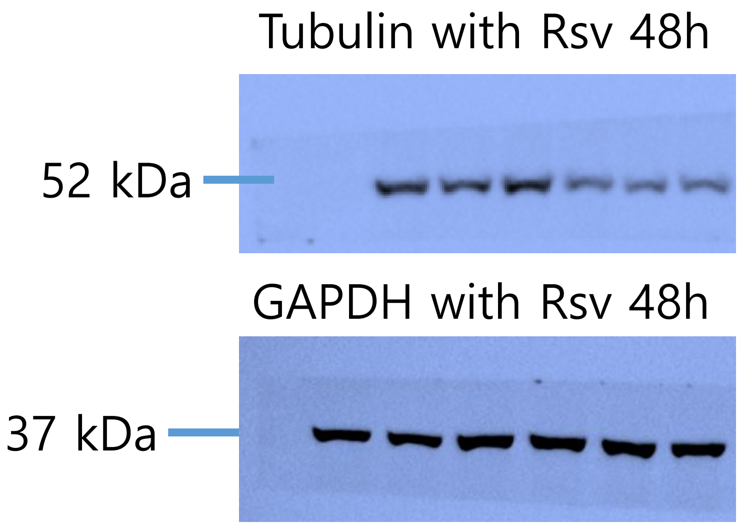

Band 1: Ctl
Band 2: Rsv 40 μM
Band 3: ISO 5 μM
Band 4: ISO 10 μM
Band 5: ISO 20 μM
Band 6: ISO 40 μM

Figure 2 original data

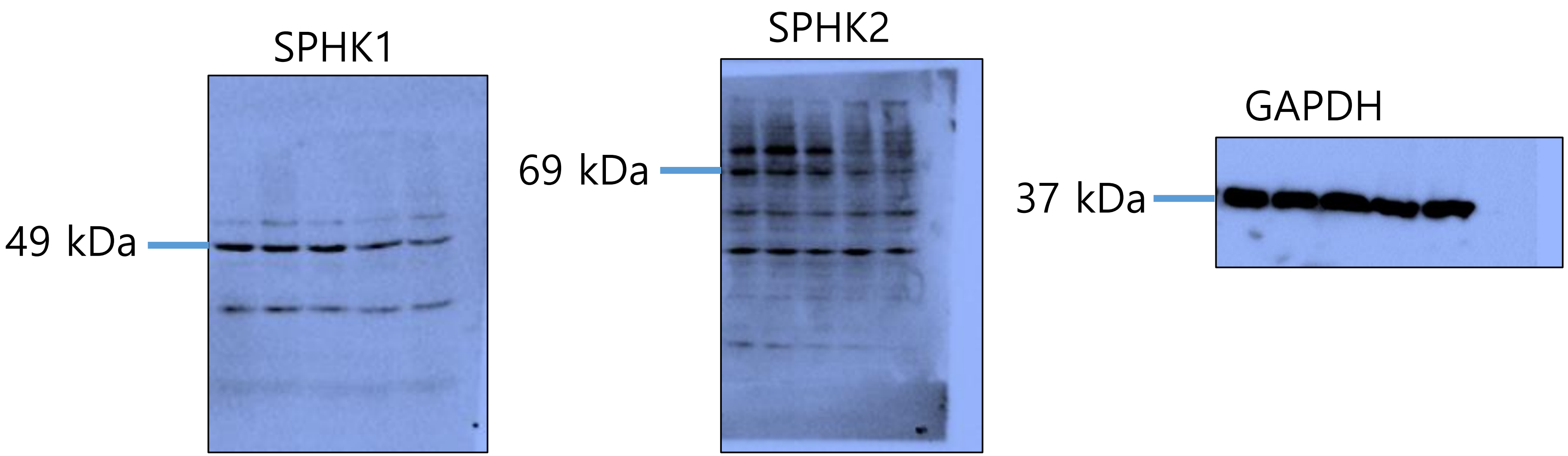

Band 1: Ctl
Band 2: ISO 5 μM
Band 3: ISO 10 μM
Band 4: ISO 20 μM
Band 5: ISO 40 μM

Note: For Manuscript we used only Ctl, ISO 20 μM and ISO 40 μM

Figure 4 original data

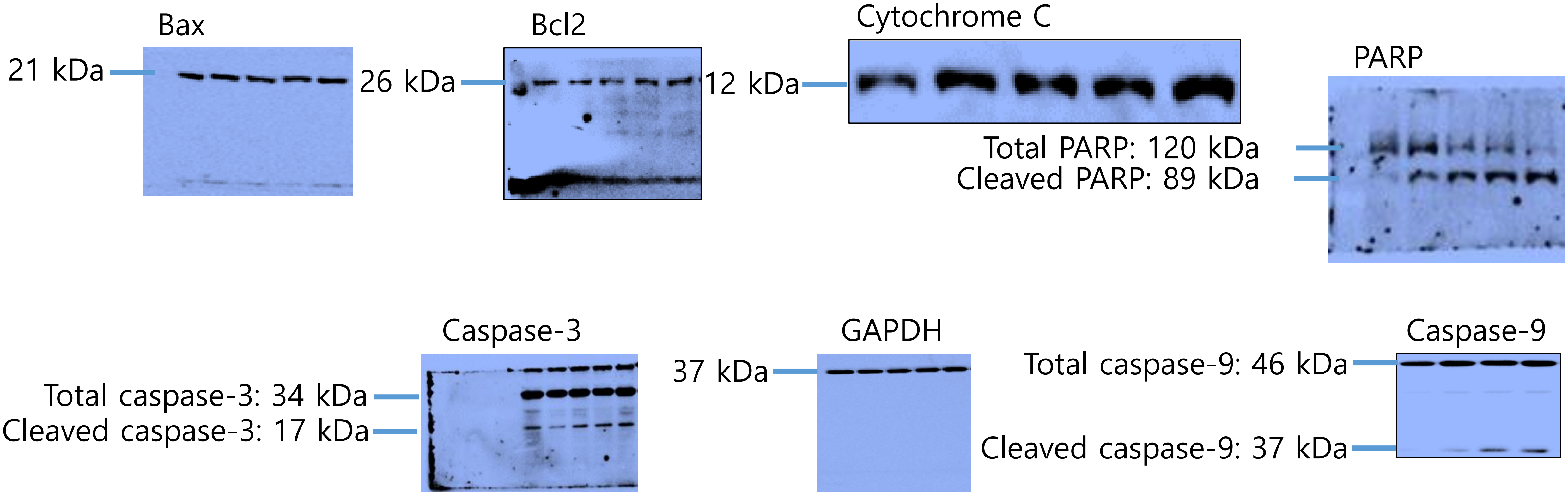

Bax, Bcl2, Cytochrome C, PARP, Caspase-3, GAPDH

Band 1: Ctl
Band 2: ISO 5 μM
Band 3: ISO 10 μM
Band 4: ISO 20 μM
Band 5: ISO 40 μM

Caspase-9

Band 1: Ctl
Band 2: ISO 10 μM
Band 3: ISO 20 μM
Band 4: ISO 40 μM

Note: For Manuscript we used only Ctl, ISO 20 μM and ISO 40 μM

Figure 5 original data

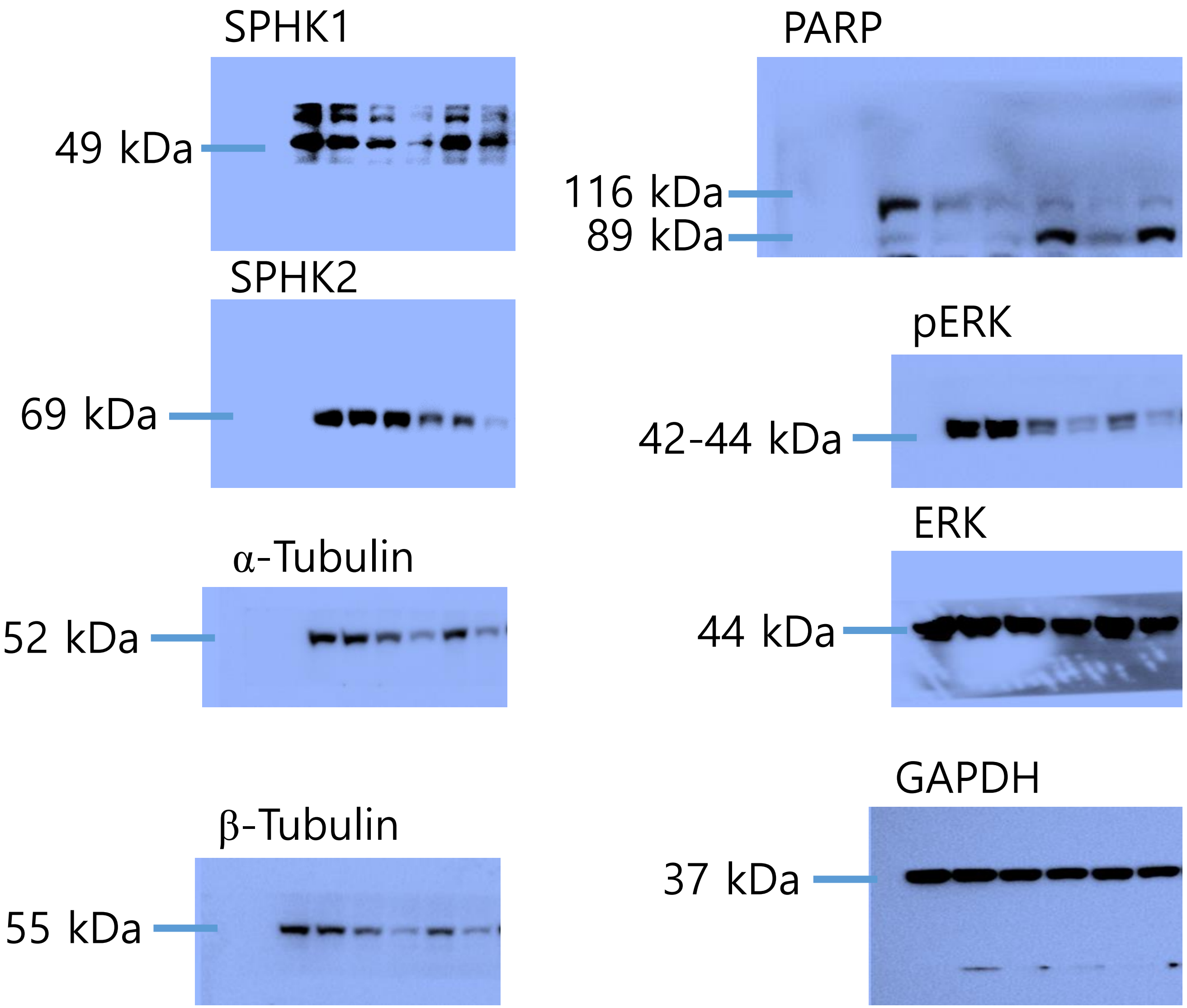

Band 1: Ctl
Band 2: NC siRNA
Band 3: SPHK1^{-}
Band 4: SPHK1^{-}+ ISO 40 μM
Band 5: SPHK2^{-}
Band 6: SPHK2^{-}+ ISO 40 μM

Figure 7 original data

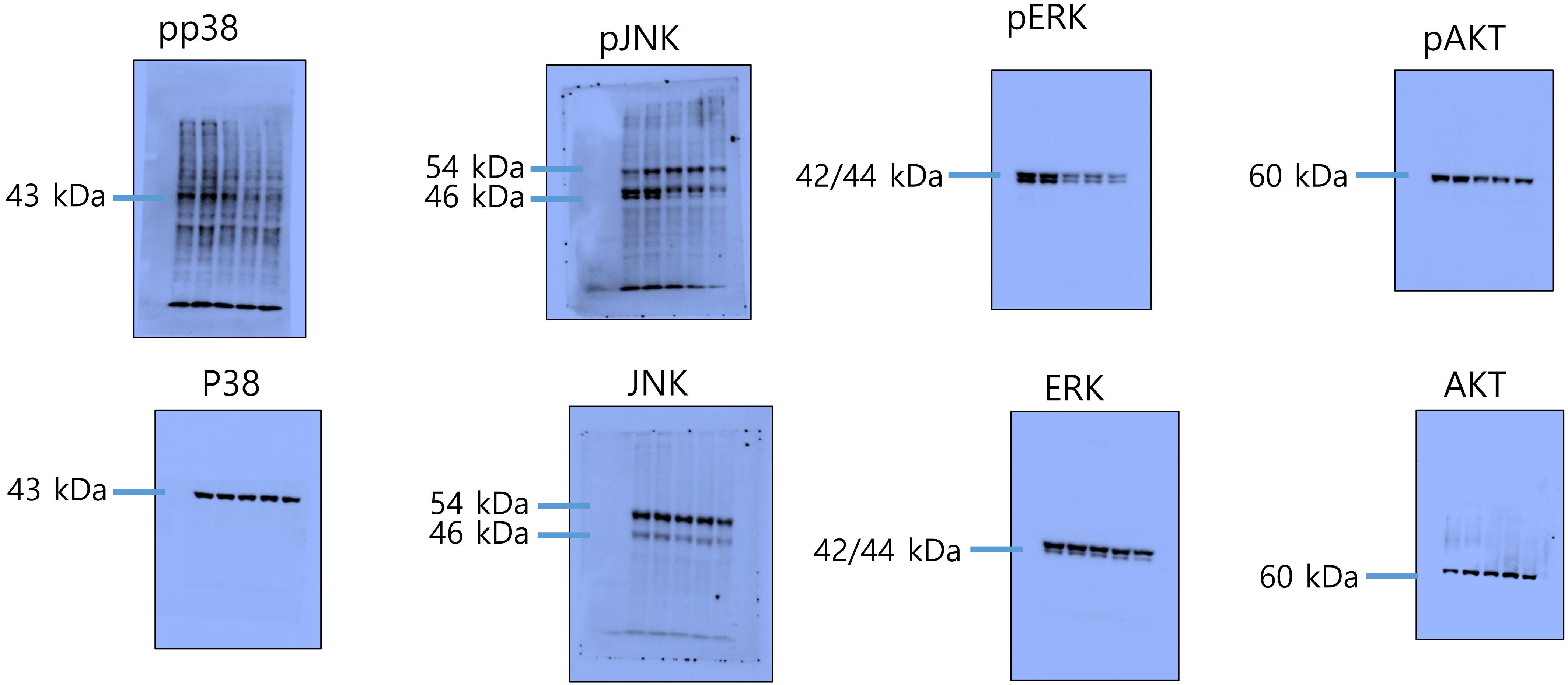

Band 1: Ctl   Band 2: ISO 5 μM
Band 3: ISO 10 μM   Band 4: ISO 20 μM
Band 5: ISO 40 μM

Note: For Manuscript we used only Ctl, ISO 20 μM and ISO 40 μM

Figure 9 original data

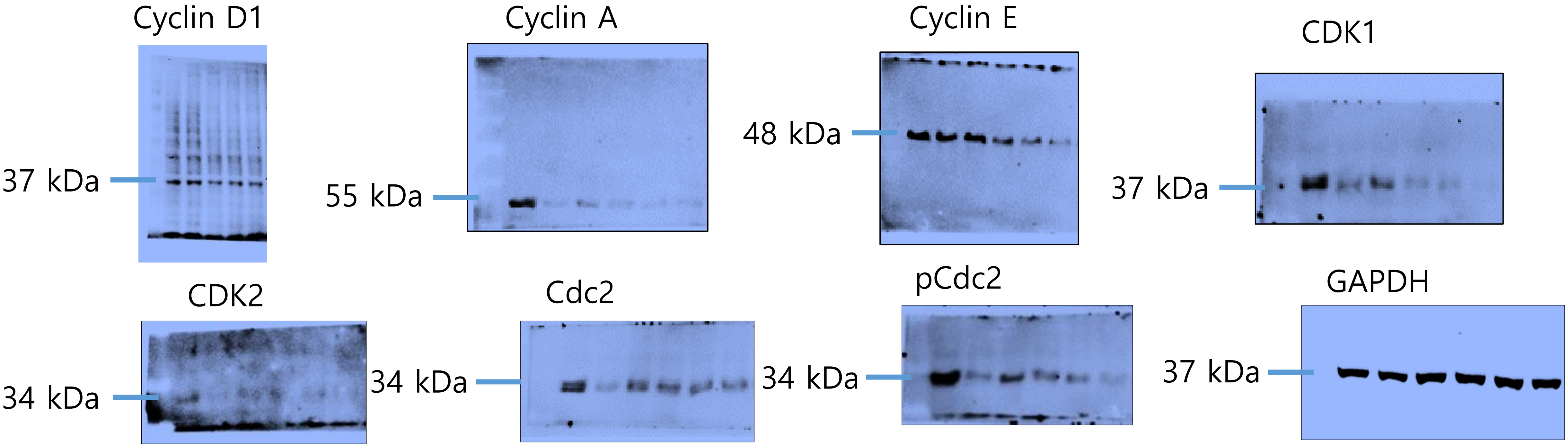

Cyclin D1

Band 1: Ctl
Band 2: Rsv 40 μM
Band 3: ISO 10 μM
Band 4: ISO 20 μM
Band 5: ISO 40 μM

Cyclin A, Cyclin E, CDK1, CDK2, cdc2, pcdc2, GAPDH

Band 1: Ctl
Band 2: Rsv 40 μM
Band 3: ISO 5 μM
Band 4: ISO 10 μM
Band 5: ISO 20 μM
Band 6: ISO 40 μM

Note: For Manuscript we used only Ctl, Rsv 40 μM, ISO 20 μM, and ISO 40 μM

Figure 10 original data

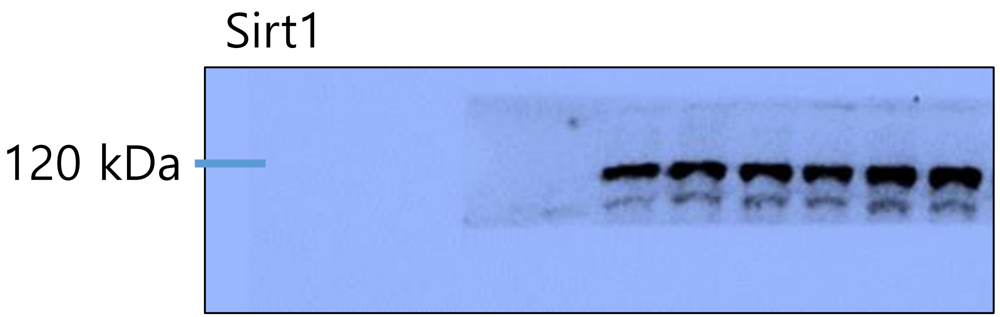

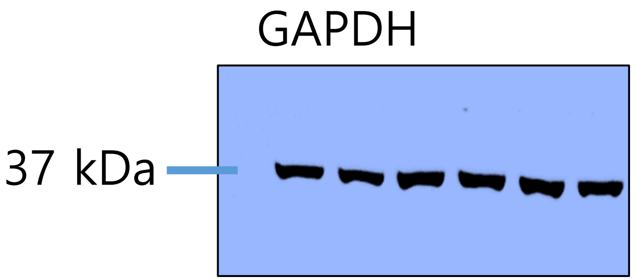

Band 1: Ctl
Band 2: Rsv 40 μM
Band 3: ISO 5 μM
Band 4: ISO 10 μM
Band 5: ISO 20 μM
Band 6: ISO 40 μM

Figure 12 original data

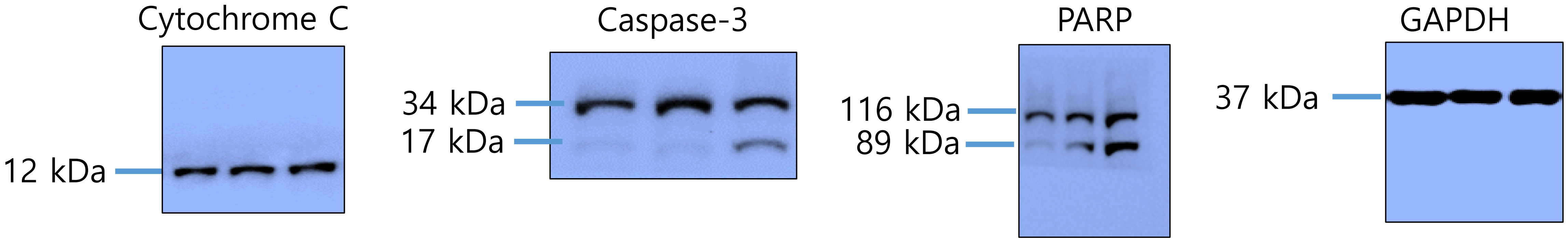

Original blot for Figure 12 (F)

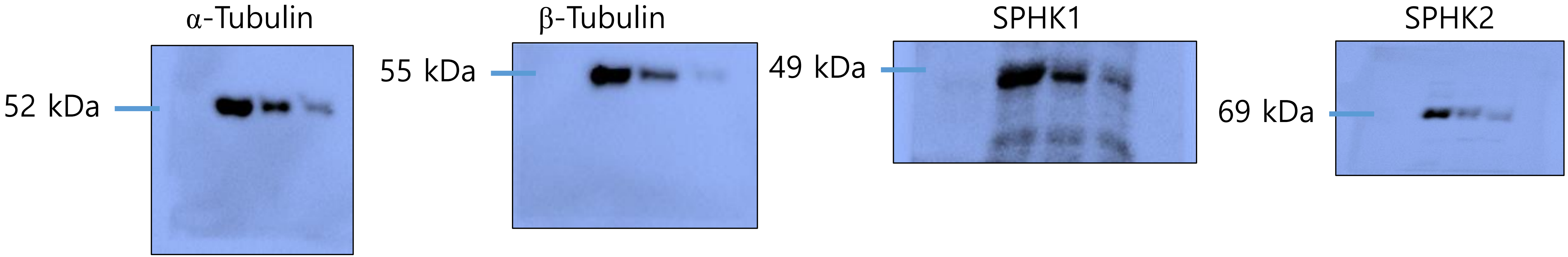

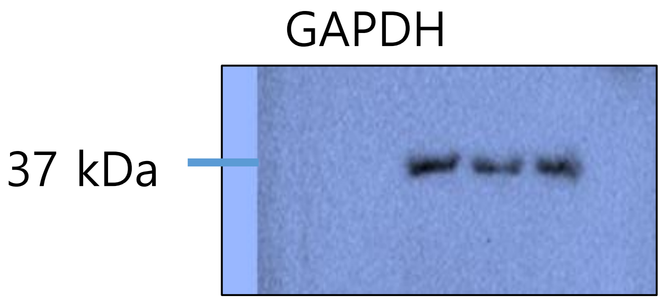

Original blot for Figure 12 (G)

Band 1: Ctl
Band 2: ISO 20 μM
Band 3: ISO 40 μM
